# Targeted photodynamic therapy for breast cancer: the potential of glyconanoparticles†

**DOI:** 10.1039/d3na00544e

**Published:** 2023-10-27

**Authors:** Brydie A. Thomas-Moore, Simone Dedola, David A. Russell, Robert A. Field, María J. Marín

**Affiliations:** a Iceni Glycoscience Ltd. Norwich Research Park Norwich NR4 7TJ UK m.marin-altaba@uea.ac.uk; b School of Chemistry, University of East Anglia Norwich Research Park Norwich NR4 7TJ UK; c Department of Chemistry and Manchester Institute of Biotechnology, University of Manchester 131 Princess Street Manchester M1 7DN UK

## Abstract

Photodynamic therapy (PDT) uses a non-toxic light sensitive molecule, a photosensitiser, that releases cytotoxic reactive oxygen species upon activation with light of a specific wavelength. Here, glycan-modified 16 nm gold nanoparticles (glycoAuNPs) were explored for their use in targeted PDT, where the photosensitiser was localised to the target cell through selective glycan–lectin interactions. Polyacrylamide (PAA)-glycans were chosen to assess glycan binding to the cell lines. These PAA-glycans indicated the selective uptake of a galactose-derivative PAA by two breast cancer cell lines, SK-BR-3 and MDA-MD-231. Subsequently, AuNPs were modified with a galactose-derivative ligand and an amine derivate of the photosensitiser chlorin e6 was incorporated to the nanoparticle surface *via* amide bond formation using EDC/NHS coupling chemistry. The dual modified nanoparticles were investigated for the targeted cell killing of breast cancer cells, demonstrating the versatility of using glycoAuNPs for selective binding to different cancer cells and their potential use for targeted PDT.

## Abstract 2

Glyconanoparticles selectively bind to cellular targets *via* glycan–lectin interactions and are excellent drug-delivery systems for targeted photodynamic therapy of cancer.

## Graphical abstract

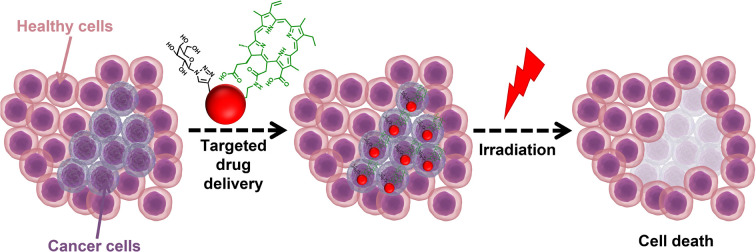

## 1. Introduction

Glycans, including glycolipids and glycoproteins, form a cell-surface coating around the outermost membrane of a cell, known as the ‘glycocalyx’.^1^ Cell-surface glycans are ‘read’ by proteins expressed by cells and the interactions play critical roles in a number of biological processes,^2^ including cellular attachment, recognition, signalling, embryonic development, growth, metastasis, and differentiation. A predominant group of glycan-binding proteins are ‘lectins’.^3^ The glycan–lectin interaction is highly selective for saccharides, similar in selectivity to antibody–antigen and enzyme–substrate interactions.^4^ Lectins rely on specific protein modules for glycan binding, called the carbohydrate recognition domain (CRD) and, since the glycan–lectin interaction is relatively weak, lectins rely on multivalent binding achieved by multiple CRDs to strengthen these interactions,^5^ exploiting the multivalent carbohydrate presentation of the glycocalyx.

Glycan–lectin interactions are involved in a wide variety of processes in the human body, and lectin expression can be altered in diseased-state cells. For example, in cancer the glycan–protein interactions play key roles in avoiding immunosurveillance and reattachment to new tissue during metastasis.^6^ Cancer cells are also associated with increased metabolism due to their unregulated, increased growth,^7^ reflected in an increase of glycan transporters. For breast cancer cells, there are key lectins and glycan-binding receptors that are upregulated, which include galectins (lectins that bind β-d-galactosides),^8,9^ glucose transporters (GLUTs) (responsible for glucose uptake but have also shown selectivity for other hexoses, such as galactose and fructose),^10,11^ and the mannose receptor.^12,13^ Consequently, galactose, glucose, lactose, and mannose are often assessed for targeted binding of glycoconjugates to cancer cell lines and thus, for the targeted delivery of imaging agents or drugs to breast cancer cells.^14^

The use of drug delivery systems can enhance safety and efficacy of the therapeutic^15^ by specifically delivering the drug to the diseased or infected area at a controlled rate.^16^ In order to combine targeting and eradication elements, a ‘carrier’ is often used. Many types of carriers have been developed for drug delivery over the past thirty years,^15^ and in particular, nanoparticles (NPs) have gained significant interest. Amongst the different types of nanoparticles reported in the literature, gold NPs (AuNPs) have been studied in great detail since they offer superior control over NP size and shape, and consequently monodispersity.^17^ The relative ease of AuNP surface modification, together with their unique optical properties, has amplified interest in the application of AuNPs as drug carriers, as well as in cellular imaging and sensing.^18^ For example, by coating the AuNP surface with glycans (glycoAuNPs), binding to their corresponding lectin can be observed through colour changes. This is a result of multivalent glycoAuNPs binding to multimeric lectins, creating an ‘aggregate’ of particles; a concept that has been explored for the detection of soluble lectins,^19^ toxins,^20^ bacterial cells,^21^ and viruses.^22^

GlycoAuNPs have been explored in therapeutic applications by, for example, coupling glycans and known drugs to the surface, aiming to improve delivery and efficacy of a licensed drug. In glycan targeting, the AuNPs display multiple copies of the glycan-based targeting agents, which renders the multivalent presentation required to increase the binding to the target (lectin). Some systems use polysaccharide-coated AuNPs successively conjugated to conventional drugs. For example, Jang *et al.* demonstrated improved delivery of the anticancer drug doxorubicin by coating the AuNP surface with dextran, and then conjugating doxorubicin to the dextran coat.^23^ The results showed high toxicity to HeLa cells at low concentrations of doxorubicin conjugate particles, in comparison to the unconjugated doxorubicin. Monosaccharides have also been used for targeted drug delivery using AuNPs. Conde *et al.* used a multicomponent system for AuNP functionalisation involving glucose, siRNA to target a gene involved in modulating apoptosis and proliferation, polyethylene glycol (PEG), and a fluorophore.^24^ The study showed promising results against lung cancer *in vitro* and *in vivo* (mice), with an 80% reduction in tumour size *in vivo*.

One type of therapy that has been employed for anti-cancer treatment through NPs is photodynamic therapy (PDT), which relies on a non-toxic photosensitiser (PS) that, upon light activation with a suitable light source, releases reactive oxygen species (ROS) that cause irreversible damage to cells where the PS is localised.^25^ PDT is particularly attractive to treat skin cancer and tumours located close to the skin surface, such as breast cancer.^26,27^ Without suitable light activation, the PS is harmless to the cell. Once the PS is irradiated, the half-life of the singlet oxygen toxic species is short (<40 ns) and restricted to a small area (<20 nm),^28^ reducing collateral damage to healthy cells and tissues. Precise targeting of the appropriate cancerous cells is therefore a critical step to efficiently deliver the PS selectively. The PS drugs used in PDT are often hydrophobic thus, coupling them to NPs can improve the dispersibility and delivery.^29^ The NPs can also offer multi-copy ligand presentation, and so, a targeting element can be introduced with the PS.^30–33^ Expression of carbohydrate receptors on the surface of cancer cells can be exploited for targeting. The hydrophobicity of PSs often translates into poor solubility *in vivo* or formation of aggregates that lowers their efficacy, or can lead to quenching. As glycans offer hydrophilicity when conjugated to compounds, there has been much research into using glycan–PS conjugates for improving biocompatibility, as well as targeting. Research using this approach focused on galactose-, glucose-, mannose-, and lactose-based PS conjugates,^34–40^ and research papers and reviews describing the use of glyco-modified PSs have been published.^41–44^

Our group has previously developed targeted PDT of cancer cell lines exploiting the glycan–lectin interaction for targeting and AuNPs as a scaffold.^31,45^ Obaid *et al.* demonstrated targeted PDT using 4 nm AuNPs and the plant lectin: jacalin.^45^ The lectin binds to Galβ1-3GalNAc residues (Thomsen–Friedenreich or ‘T’ antigen) that are expressed by 90% of primary human carcinomas; whereas, in normal tissue, T antigens are usually hidden to the immune system, and not exposed on the cell surface.^46^ The results demonstrated selective, glycan–lectin targeted PDT of HT-29 colon cancer cells, highlighting the potential of using this system for targeted PDT. There are advantages to modifying the drug with a glycan rather than a lectin. Natural lectins are large biomolecules that can be immunogenic and toxic.^47^ It is also much more difficult to obtain pure material with a lectin as they are usually extracted from biological material. Furthermore, recombinant lectins are often low yielding and high cost to obtain as pure material.^48^ Using glycans in drug conjugates for targeting also provides additional advantages since they offer improved aqueous solubility, biocompatibility, and established synthetic strategies to generate purified compounds. Using this approach, García Calavia *et al.* generated *ca.* 4 nm AuNPs functionalised with a phthalocyanine derivative (C3Pc) and a lactose derivative to target galectin-1 (a β-galactose-binding lectin that has been documented as being overexpressed in breast cancer), and tested the construct against two breast cancer cell lines: MDA-MB-231 and SK-BR-3.^31^ These studies demonstrated selective cell death of the SK-BR-3 cell line with the lactose-C3Pc-AuNPs after 3 hours incubation; but no selective cell death was observed for the MDA-MB-231 cell line after 3 hours. The improved cytotoxicity for the SK-BR-3 cell was unexpected as lower galectin-1 expression was detected, compared to the MDA-MB-231. The authors speculated that a second lactose-binding protein, GLUT1, could be involved in the AuNP uptake. The results became further complexed with regard to the MDA-MB-231 cell line, as a galectin-1 inhibition assay showed selective binding to galectin-1 after 3 hours incubation of lactose-C3Pc-AuNPs; whereas, no cell death was observed with the same conditions in the PDT studies. Similarly, Durand and co-workers reported mannose-functionalised mesoporous silica nanoparticles for efficient targeted PDT of breast cancer cells.^49^ A 99% cell death was reported with the mannose-conjugated particles upon irradiation of the PS drug, while only a 45% cell death was achieved without mannose, demonstrating the mannose-dependent interaction. Subsequently, the authors further explored the potential of mannose receptors targeting to achieve efficient two-photon PDT of colorectal solid tumours using mannose-functionalised mesoporous silica nanoparticles;^50^ and reported the superior targeting ability of dimannoside derivatives over monosaccharides for the efficient PDT of prostate cancer.^51^

**Fig. 1:**
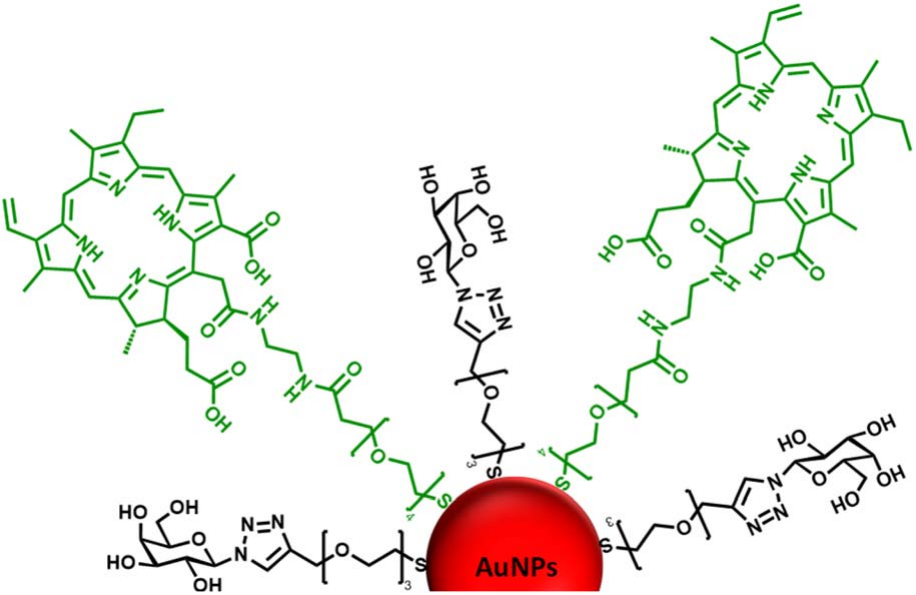
Schematic representation of the *ca.* 16–17 nm AuNPs (red) functionalised with a galactose derivative ligand (black) and a chlorin e6 derivative ligand (green).

Considering the previous results from our group^31^ and those reported by others,^49–51^ identifying a glycan candidate through glycan binding studies could improve the selective cell killing of cancer cells lines *via* PDT using NPs. Here, glycan binding to two breast cancer cell lines, and to a noncancerous breast epithelial cell line, was assessed following an innovative protocol through commercial polyacrylamide (PAA) probes. The PAA contained biotin along with one of the following glycans (PAA-glycans): galactose (PAA-Gal), glucose (PAA-Glc), lactose (PAA-Lac), or mannose (PAA-Man). The direct binding between PAA-glycan to the breast cell lines was assessed evaluating the cellular uptake using confocal microscopy. The results demonstrated different selectivity of each glycan towards the cell lines, with galactose showing statistically significant uptake by both cancer cell lines, but not to the non-cancer cell line. These results informed our PDT studies as AuNPs were functionalised with a galactose derivative ligand and the PS chlorin e6 (ce6) (see schematic representation of the glycoAuNPs in Fig. 1). The potential to achieve targeted cell killing of breast cancer cells *via* PDT was investigated. All the previous studies by our group have been performed successfully using 4 nm AuNPs. With consideration of the aqueous solubility that the glycan provides, for this present study larger AuNPs, *ca.* 16–17 nm, synthesised with citrate as the reductant, were used. The uptake of AuNPs by overexpressed breast cancer glycan-binding proteins was assessed using inhibition assays and confocal microscopy.

## 2. Materials and methods

Further details on materials, buffers, and media used; instrumental techniques; and cell culturing methods can be found in the ESI.†

### 2.1 Biotinylated-polyacrylamide-glycan binding to cell lines

Dislodged cells were centrifuged to remove trypsin and corresponding media. Breast cancer cell lines, MDA-MB-231 or SK-BR-3, were resuspended in Dulbecco's Modified Eagle Medium (DMEM) without foetal bovine serum (FBS), DMEM(−), while the healthy breast epithelial cells, MCF-10A cells, were resuspended in Mammary Epithelial Cell Growth Medium (MEGM). The cells were counted and diluted to a working concentration of 1 × 10^4^ cells per mL. To Nunc 6-well multidishes, an 18 mm diameter glass coverslip was placed in each well. To each well, 2 mL of cell culture was added and incubated for 24 hours at 37 °C in a 5% CO_2(atmosphere)_. The culture media was discarded, and washed with 3 mL of PBS, twice. A 40 μg mL^−1^ of each PAA-glycan was prepared in either DMEM(−) (MDA-MB-231 or SK-BR-3) or MEGM (MCF-10A), and 1 mL was added per well. The cells were incubated for 1 hour at 37 °C in a 5% CO_2(atmosphere)_. The media was then discarded, and the coverslips were washed twice with 3 mL of phosphate buffer saline (PBS). Then, Alexa Fluor 488-labelled streptavidin (AF488-st, 10 μg mL^−1^) was added to each well and incubated for 30 min, at 37 °C in a 5% CO_2(atmosphere)_. The wells were then washed twice with 3 mL of PBS. Finally, 1 mL of BioTracker Orange dye (0.83 μg mL^−1^) was added to the cells and incubated for 30 min at 37 °C in a 5% CO_2(atmosphere)_. The wells were then washed with PBS, three times, and resuspended in 1 mL of DMEM with FBS (DMEM(+)) or MEGM.

For confocal microscopy imaging, the coverslips were held in place with a Ludin chamber. The coverslip was washed three times with imaging medium (1 mL), and 1 mL of imaging medium was added to the cover slip for imaging. A heating stage was used to hold the Ludin chamber in the confocal microscope, and heated to 37 °C. The AF488-st was excited with a 488 nm argon-ion laser (emission collected from 505–530 nm), and the Biotracker Orange dye was excited with a 514 nm argon-ion laser (emission collected at wavelengths >550 nm). Differential interference contrast (DIC) images were collected alongside the fluorescence imaging. Controls with PAA (no glycan), just cells (no dyes), and cells with just AF488-st (no PAA-glycan), were also imaged to assess non-selective binding and any autofluorescence from the cells.

### 2.2 Synthesis and characterisation of glycan ligands and functionalised gold nanoparticles

#### 2.2.1 Synthesis of azide 1 and alkyne 2

Azide (1) was synthesised following a method reported by Tropper *et al.*^52^ while alkyne (2) was synthesised following a method reported by Goswami *et al.*^53^

#### 2.2.2 Synthesis of β-d-galactosyl triazole 3

Azide (1) (33 mg, 88.5 μmol) and alkyne (2) (28 mg, 113.8 μmol, 1.1 equiv.) were each dissolved in 500 μL of dimethylformamide (DMF) and mixed together. In ultra-pure water, 1 M solutions of CuSO_4_, sodium ascorbate (NaAsc) and tris-hydroxypropyltriazolylmethylamine (THPTA) were prepared. A premixed solution of CuSO_4_ (17.7 μmol, 17.7 μL, 0.2 equiv.) and THPTA (44.2 μmol, 44.2 μL, 0.5 equiv.) was prepared and then added to the reaction mixture, followed by NaAsc (35.4 μmol, 35.4 μL, 0.4 equiv.). The reaction was heated to 50 °C and stirred for 16 hours. The reaction was monitored by thin layer chromatography (TLC) and Advion ESI-MS (positive mode). The DMF was evaporated under vacuum and the reaction mixture dissolved in dichloromethane (DCM) : H_2_O (9.5 : 0.5) for organic/aqueous phase extraction. The organic phase (DCM) was collected, dried over MgSO_4_, filtered and then solvent evaporated under vacuum. The product was purified by silica gel chromatography (hexane : ethyl acetate (Hex : EtOAc) 9.5 : 0.5 → 2.5 : 7.5) to give compound 3 (Fig. S1a†), Ac-galactose-PEG_3_-SAc, as an oil (45.6 mg, 84.9%); R_*f*_ 0.44 (100% EtOAc); [*α*]_D_ −2.5 (*c* 1.0, 20 °C, CHCl_3_); *δ*_H_ (400 MHz, CDCl_3_); 7.85 (s, 1H, H-a), 5.85 (d, 1H, H-1), 5.60–5.50 (m, 2H, H-3, H-4), 5.25 (dd, 1H, H-2), 4.64 (s, 2H, CH_2_), 4.28–4.06 (m, 3H, H-5, H-6, H-6′), 3.68–3.55 (m, 10H, 5(CH_2_)), 3.08 (m, 2H, CH_2_), 2.32 (s, 3H), 2.22 (s, 3H), 2.16 (s, 3H), 2.06–1.97 (m, 7H), 1.88 (s, 3H), 1.31–1.20 (m, 2H), 2.26 (s, 3H, CH_3_), 2.16 (s, 3H, CH_3_), 1.98 (s, 3H, CH_3_), 1.94 (s, 3H, CH_3_), 1.82 (s, 3H, CH_3_); *δ*_C_ (600 MHz, CDCl_3_) 171.1, 170.3, 169.9, 169.8 and 169.0 (COCH_3_), 145.9 (C-b), 121.3 (C-a), 86.4 (C-1), 74.1 (C-5), 70.8 (C-2), 70.51, 70.49, 70.3 and 69.8 (CH_2_), 67.9 (C-3), 66.8 (C-4), 64.5 (CH_2_), 61.3 (C-6), 60.3 (CH_2_), 30.5 (CO**C**H_3_), 28.7 (CH_2_), 21.0, 20.6, 20.5 and 20.2 (COCH_3_); HR ESI-MS found *m*/*z* 641.7872 [M + Na]^+^ calculated for C_25_H_37_N_3_O_13_SNa 642.1939.

#### 2.2.3 Synthesis of galactosyltriazole-PEG_3_-SH (Gal-PEG_3_-SH) 4

Compound (3) (21.2 mg, 20.2 μmol) was dissolved in anhydrous methanol (4.5 mL). Under nitrogen, sodium methoxide (0.02 M, 5 equiv.) was added and stirred at room temperature for one hour, until only deacetylated compound could be detected. The reaction was monitored by ESI MS and NMR. Amberlite 120H^+^ resin was added to neutralise the reaction mixture, and then filtered. The solvent was removed under vacuum. The product was purified by GPC, using a Toyopearl TSK-HW40S column (60 cm, 0.5 mL min^−1^, H_2_O), and collected between 220–240 min. The purified product compound 4 (Gal-PEG_3_-SH, Fig. S1b†) was a fine powder (11.4 mg, 81.4%); [*α*]_D_ +15.2 (*c* 1.0, 20 °C, H_2_O); *δ*_H_ (400 MHz, D_2_O) 8.20 (s, 1H, H-a), 5.60 (d, 1H, *J*_1,2_ = 9.51 Hz, H-1), 4.63 (s, 2H, H-c), 4.12 (t, 1H, *J*_1,2_ = 9.51, *J*_2,3_ = 9.51, H-2) 3.98 (d, 1H, *J*_3,4_ = 3.34, H-4), 3.91 (m, 1H, H-5), 3.78 (dd, 1H, *J*_2,3_ = 9.51, *J*_3,4_ = 3.34, H-3), 3.71–3.52 (m, 12H, H-6, CH_2_) 2.60 (t, 2H, H-i); *δ*_C_ (400 MHz, D_2_O) 144.13 (C-b), 124.09 (C-a), 87.96 (C-1), 78.23 (C-5), 72.85 (C-3), 72.10 (C-h), 69.62 (C-2), 69.47, 69.42, 69.10, 68.95 (C-d/C-e/C-f/C-g), 68.47 (C-4), 62.92 (C-c), 60.75 (C-6), 22.98 (C-i); HR ESI-MS found *m*/*z* 817.2931 [M + H]^+^ calculated for C_30_H_52_N_6_O_16_S_2_H (disulphide product) 817.2954.

#### 2.2.4 Functionalisation of AuNPs with galactose-PEG_3_-SH ligand

16 nm citrate stabilised gold nanoparticles (citrate-AuNPs) were prepared following the method developed by Turkevich *et al.*^54^ A 10 mM (4 mg mL^−1^) Gal-PEG_3_-SH solution was prepared in ultra-pure water. To vials containing 9.8 mL of 3 nM citrate-AuNPs, 200 μL of the 10 mM stock solution was added, to provide a total volume of 10 mL, and final Gal-PEG_3_-SH concentrations of 200 μM. The solutions were stirred for 72 hours at room temperature. The suspensions were transferred to spin columns (10 kDa MW cut-off) and centrifuged at 4000 × *g* for 10 min. The concentrated Gal-PEG_3_-SH functionalised AuNPs suspension (Gal-PEG_3_-AuNPs) were diluted in 10 mL of ultra-pure water and centrifuged for a further 10 min at 4000 × *g*. The process was then repeated two more times to remove all the unbound ligand. The extinction spectrum of the purified Gal-PEG_3_-AuNPs was recorded in a plate reader, and MALDI-TOF MS was used to detect the Gal-PEG_3_-SH on the nanoparticles.

#### 2.2.5 Functionalisation of AuNPs with mixed monolayer of chlorin e6 and glycan ligand

Stock solutions of HOOC-PEG_4_-SH (2.8 mg mL^−1^) and Gal-PEG_3_-SH (4 mg mL^−1^) were prepared in ultra-pure water to provide a final concentration of 10 mM. To a vial containing 9.8 mL of 3 nM citrate-AuNPs, 50 μL of HOOC-PEG_4_-SH and 150 μL of Gal-PEG_3_-SH stock solutions (10 mM) were added, to provide final concentrations of the HOOC-PEG_4_-SH and Gal-PEG_3_-SH at 50 μM and 150 μM, respectively. The suspension was stirred for 72 hours. To purify the suspension and remove excess ligand, the solution was transferred to spin columns (10 kDa MW cut-off) and centrifuged at 4000 × *g* for 10 min. The concentrated Gal-PEG_3_-SH and HOOC-PEG_4_-SH functionalised AuNP suspension (Gal-PEG_3_-/COOH-PEG_4_-AuNPs) was diluted in 10 mL of ultra-pure water and centrifuged for a further 10 min at 4000 × *g*. The process was then repeated two more times.

*N*-Hydroxysuccinimide (NHS, 3.5 mg mL^−1^) and 1-ethyl-3-(3-dimethylaminopropyl)carbodiimide hydrochloride (EDC, 5.8 mg· mL^−1^) stock solutions were prepared in ultra-pure water, to provide a final concentration of 30 mM. In a fresh vial, purified Gal-PEG_3_-/COOH-PEG_4_-AuNPs were diluted in 4-morpholinoethanesulfonic acid (MES) buffer to provide a final volume of 9.9 mL. To the Gal-PEG_3_-/COOH-PEG_4_-AuNPs, 50 μL of the 30 mM EDC and NHS stocks were added, to provide a final concentration of 150 μM. The reaction mixture was stirred for 2 hours at room temperature. To purify the suspension and remove excess reagents, the suspension was transferred to spin columns (10 kDa MW cut-off) and centrifuged at 4000 × *g* for 10 min. The concentrated Gal-PEG_3_-SH and NHS activated functionalised AuNP suspension (Gal-PEG_3_-/NHS-PEG_4_-AuNPs) was diluted in 10 mL of ultra-pure water and centrifuged for a further 10 min at 4000 × *g*. The process was then repeated two more times.

A 73 mM stock solution of the ce6-NH_2_ derivative (50 mg) was prepared in 1 mL of methanol (MeOH). In a fresh vial, purified Gal-PEG_3_-/NHS-PEG_4_-AuNPs were diluted in phosphate buffer (PB) to provide a final volume of 9.9795 mL. To the Gal-PEG_3_-/NHS-PEG_4_-AuNPs, 20.5 μL of the ce6-NH_2_ stock solution was added, to provide a final concentration of 150 μM. The reaction mixture was stirred for 16 hours at room temperature, in the dark. To purify the suspension and remove excess reagents, the suspension was transferred to spin columns (10 kDa MW cut-off), and centrifuged at 4000 × *g* for 10 min. The concentrated Gal-PEG_3_-SH and ce6-PEG_4_-SH functionalised AuNP suspension (Gal-PEG_3_-AuNPs/ce6-PEG_4_-AuNPs) was diluted in 10 mL of ultra-pure water and centrifuged for a further 10 min at 4000 × *g*. The process was then repeated six more times until all the unbound ce6 was removed. The purified suspension was characterised using MALDI-TOF- MS, and the extinction spectrum was recorded using the plate reader.

The process was repeated with HO-PEG_3_-SH to provide PEG_3_-AuNPs/ce6-PEG_4_-AuNPs as a control.

The concentration of ce6 on the nanoparticles was calculated using the Beer–Lambert Law and 1.3 × 10^5^ M^−1^ cm^−1^ as the extinction coefficient for ce6 at 405 nm. The absorption intensity due to the ce6 on the nanoparticle was calculated by subtracting the extinction due to the nanoparticle at that wavelength.

### 2.3 Singlet oxygen studies – in solution

To a quartz cuvette, 1 mL of either Gal-PEG_3_-AuNPs/ce6-PEG_4_-AuNPs, PEG_3_-AuNPs/ce6-PEG_4_-AuNPs or Gal-PEG_3_-AuNPs were added. The particles were diluted to provide ce6 concentration at 300 nM. The fluorescence was measured using a fluorimeter, with excitation wavelength at 360 nm and emission collected between 380–600 nm. A stock solution of the singlet oxygen probe 9,10-anthracenediyl-bis(methylene)dimalonic acid (ABMA) was prepared in methanol (0.512 mM). To the corresponding AuNP solution, 2 μL of ABMA was added to provide a final concentration of 1 μM. The fluorescence was measured immediately. The solution was then irradiated with a 633 nm He–Ne laser. The fluorescence was measured every 5 min for 30 min. The fluorescence emission intensity at 433 nm was plotted against time to assess singlet oxygen production. As control, the experiment was repeated but without irradiation.

### 2.4 Intracellular evaluation of the functionalised nanoparticles and potential as photodynamic therapy agents

#### 2.4.1 Uptake by cell lines

Following the same preparation as described in Section 2.1, the cells were seeded onto coverslips in Nunc 6-well multidishes as 1 × 10^4^ cells per mL. After washing with PBS, Gal-PEG_3_-AuNPs/ce6-PEG_4_-AuNPs, or PEG_3_-/ce6-PEG_4_-AuNPs were added to the wells at a ce6 solution concentration of 50 nM, in DMEM(−) or MEGM. The cells were incubated for 3 hours at 37 °C in a 5% CO_2(atmosphere)_. The cells were washed three times with PBS. 0.83 μg mL^−1^ (in DMEM(+) or MEGM) of BioTracker Orange dye was added to each well, and incubated for 30 min at 37 °C in a 5% CO_2(atmosphere)_. The cells were washed three times with 3 mL of PBS and resuspended in DMEM(+) or MEGM. The cells were imaged following the same method outlined in Section 2.1.

#### 2.4.2 Inhibiting Gal-PEG_3_-/ce6-PEG_4_-AuNP interaction with cancer cell lines

Inhibitors of SGLT (Canagliflozin), galectins (33DFTG), and GLUT (WZB117) receptors were used at a concentration of 50 μM, and prepared in DMEM(−) or MEGM. Gal-PEG_3_-/ce6-PEG_4_-AuNPs were used at ce6 solution concentration of 50 nM, prepared in DMEM(−) or MEGM.

The cells were seeded as described in Section 2.1. For these studies, only the cancer cell lines were used. The cells were washed with PBS, three times. To each well 1 mL of inhibitor solution was added and incubated for 1 hour at 37 °C in a 5% CO_2(atmosphere)_. The cells were washed with 3 mL of PBS, three times. Then following the same method as Section 2.1, the Gal-PEG_3_-/ce6-PEG_4_-AuNPs were added and imaged using confocal laser scanning microscopy.

#### 2.4.3 Photodynamic therapy studies

The cells were seeded into two, white-bottom Nunc Nunclon™ Surface 96-well microplates at a concentration of 1 × 10^4^ cells per mL, with 100 μL culture per well. The two plates were incubated at 37 °C and 5% CO_2_ atmosphere for 24 hours. The wells were then washed three times with 200 μL PBS. Different concentrations of Gal-PEG_3_-/ce6-PEG_4_-AuNP suspensions were prepared in DMEM(−) or MEGM, and added to the cells (50 μL). The plates were incubated for 3 hours at 37 °C and 5% CO_2_ atmosphere. The media was then discarded and washed three times with 200 μL PBS. DMEM(+) or MEGM was then added to the wells (100 μL).

Both plates were kept at room temperature, with one of the plates taken forward for irradiation whilst the other was kept in the dark (non-irradiated). For irradiation, a 633 nm 10 mW HeNe laser, that had a biconvex diverging lens, was held 50 cm above the plate, and each well was irradiated for 6 min. This provided a total light dose of 10.5 J cm^−2^, and an irradiance of 29 mW cm^−2^. After irradiation, both plates were placed back into the incubator for 48 hours. To measure cell viability, the Cell Titre Blue Cell Viability assay kit was used. To each well 20 μL of reagent was added to both the irradiated and non-irradiated plate. The plates were shaken at 200 rpm for 10 s, before being incubated for 4 hours at 37 °C in a 5% CO_2_ atmosphere. The plates were then shaken for 10 s at 200 rpm, and then the fluorescence was measured using a plate reader, with excitation at 561 nm and emission collected at 594 nm.

## 3. Results

### 3.1 Assessing glycan binding using glycan functionalized polyacrylamide polymers

Glycan binding and subsequent uptake were assessed using two breast adenocarcinoma cell lines (MDA-MB-231 and SK-BR-3), along with a non-cancerous breast epithelial cell line (MCF-10A) used as control. The MDA-MB-231 is a triple negative breast cancer (TNBC) cell line, associated with poor prognosis from being metastatic, aggressive, and with fewer treatment options available.^55^ The SK-BR-3 is HER2-positive (estrogen receptor (ER)- and progesterone receptor (PR)-negative) and is associated with the development of resistance against targeted therapies. Both types of breast cancer cells are considered aggressive, highlighting the importance of developing alternative therapies against them. For breast cancer cells, there are key lectins and glycan-binding receptors that have been identified as being upregulated, which include galectins,^8,9^ GLUTs,^10,11^ and the mannose receptor.^12,13^ It has been demonstrated^56^ that galectin-3 expression is high in TNBC, and it is important for metastasis.^57^ The mannose receptor has been demonstrated to be expressed in breast cancer but not healthy breast tissue, albeit to varying levels between different breast cancer types.^58^ On the other hand, overexpression of GLUT-1 has been associated with aggressive breast cancers^11^ and other GLUTs that have been shown to be overexpressed in breast cancer are GLUT-2,^59^ GLUT-3,^7^ and GLUT-12.^60^ Furthermore, overexpression of GLUTs is associated with increased invasiveness and poor prognosis.^61^

**Fig. 2:**
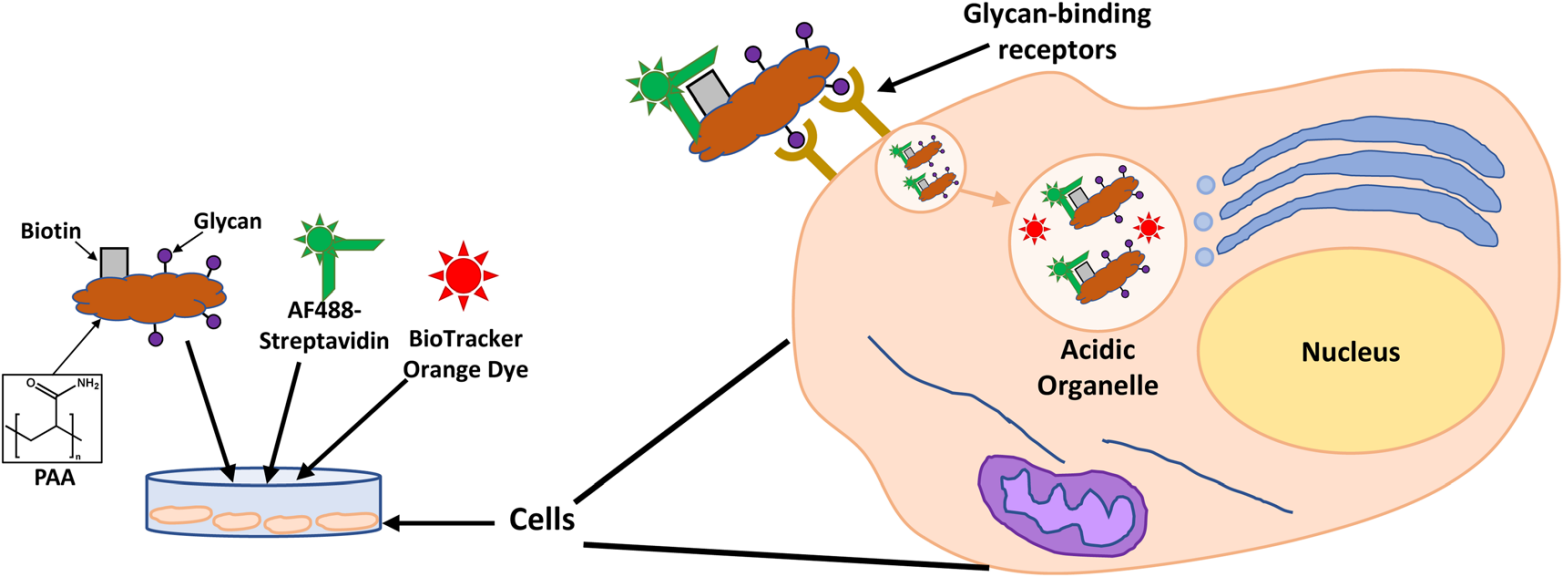
Illustration of confocal imaging studies assessing PAA-glycan interaction with cells.

With the aim of designing systems for the targeted binding to cancer cells *via* carbohydrate recognition, the first step was to screen the interaction of different glycans with the selected target cell lines to determine differences in uptake profile between cancer and non-cancer cells and therefore, to identify the best glycan candidate for selective NP targeting. For these studies, commercially available polymer glycoconjugates (from GlycoNZ) were used. The polymer was polyacrylamide (PAA) functionalised with biotin and the glycan of interest – galactose (PAA-Gal), glucose (PAA-Glc), lactose (PAA-Lac), or mannose (PAA-Man). PAA functionalised with only biotin (PAA) was used as a negative control. The cellular uptake efficiency of these PAA-glycans or control PAA by the different breast cancer cells lines was assessed using confocal microscopy. MDA-MB-231, SK-BR-3, and MCF-10A cells were treated with the corresponding PAA-glycan for a period of 1 hour and, following the removal of unbound glycan, the cells were treated with an Alexa Fluor 488 streptavidin conjugate (AF488-st) able to bind to the biotinylated-PAA-glycans that had interacted with the cells. To assess localisation of the PAA-glycans within the cells, a dye that localises in the acidic organelles (BioTracker™ 560 Orange Lysosome Dye) was used. An illustration of the confocal imaging studies is shown in Fig. 2.

**Fig. 3:**
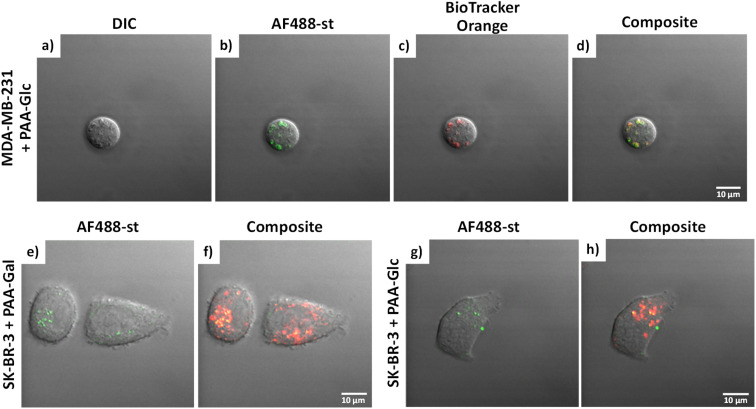
(a–d) MDA-MB-231 in the presence in PAA-Glc. Images are separated into: (a) DIC; (b) AF488-st; (c) BioTracker Orange dye; and (d) composite image. SK-BR-3 in the presence of (e and f) PAA-Gal and (g and h) PAA-Glc; images are separated into: AF488-st (green filter) (e and g) and composite image (f and h). AF488-st was imaged in the green channel using *λ*_exc_ = 488 nm and Δ*λ*_em_ = 505–530 nm; and BioTracker Orange dye was imaged in the orange channel using *λ*_exc_ = 514 nm and Δ*λ*_em_ > 550 nm. Scale bars = 10 μm.

MDA-MB-231 and SK-BR-3 breast cancer cells treated with biotin-PAA (without glycan) and AF488-st showed no detectable non-specific interaction between the PAA and the cells as no fluorescence was observed without glycan present on the PAA (Fig. S2 and S4,† respectively). Similarly, the breast cancer cell lines did not bind AF488-st when added to the cells without PAA present. Consequently, fluorescence from AF488-st was only detected when PAA-glycan was present, suggesting that the AF488-st was bound to biotin on the uptaken PAA-glycans. All glycans appear to be localising in the acidic organelles within the MDA-MB-231 cell line, as seen from the overlap between the fluorescence from the green (AF488-st) and orange filters (BioTrackerTM 560 Orange Lysosome Dye) in Fig. S2† and highlighted in Fig. 3a–d (enlarged images of MDA-MB-231 in the presence of PAA-Glc). From the confocal images, PAA-Glc appeared to have the strongest fluorescence in MDA-MB-231 breast cancer cells. In SK-BR-3 cells, PAA-Gal and PAA-Glc gave the strongest uptake result (Fig. S4†). PAA-Gal appeared to be taken up by the SK-BR-3 cells and localised in the acidic organelles as confirmed by the co-localisation between AF488-st and BioTracker orange (Fig. 3e and f). PAA-Glc also appeared to be localised in the acidic organelles, with some PAA-Glc localised in other parts of the cells, *i.e.* not co-localised with the BioTracker orange dye (Fig. 3g and h). Further investigation would be needed to determine the full localisation of the PAA-Glc across the cell, but possibly the uptake of the PAA-Glc is slower than that of the PAA-Gal, resulting in the PAA-Glc localised on the cells surface. This could be explained by the PAA-Gal and PAA-Glc interacting with different cell surface receptors, which could affect their cellular uptake rate. In the healthy cell line, MCF-10A, confocal images showed the interaction with lactose, glucose, and mannose, predominantly (Fig. S6†). In this cell line, PAA-Lac was observed to partially localise in the acidic organelles, while PAA-Man was predominantly not localised in the acidic organelles as low co-localisation between the AF488-st and BioTracker Orange was observed. For PAA-Glc, there appeared to be no localisation of the PAA-glycan within the acidic organelles. The difference in localisation between the PAA-glycans suggests different uptake pathways, whether that be the receptor involved, or rate of uptake.

Measurements of the mean integrated intensity of the emission observed in the confocal images were used to estimate the uptake of the different glycans to MDA-MB-231 (Fig. S3†), SK-BR-3 (Fig. S5†) and MCF-10A (Fig. S7†). For MDA-MB-231 breast cancer cells, the strongest interaction was observed with PAA-Lac. There was also a statistically significant interaction between PAA-Gal and PAA-Glc for MDA-MB-231. However, no statistically significant interaction was detected with PAA-Man and MDA-MB-231. From the confocal images (Fig. S2†) and from the integrated intensity (Fig. S3†), there appears to be some level of uptake of PAA-Man by MDA-MB-231 but this was not a consistent, statistically significant interaction, which is emphasised by the weaker signal in the confocal images. As observed in Fig. S5,† PAA-Gal clearly showed the greatest interaction with SK-BR-3, followed by PAA-Glc, which is consistent with the confocal images in Fig. S4.† Both PAA-Lac and PAA-Man did not show a significant interaction with SK-BR-3 cells. For MCF-10A, the strongest interaction was observed with PAA-Lac, and PAA-Glc and PAA-Man also showed significant interactions with this cell line (Fig. S7†). Comparing all the PAA-glycan results with each cell line (Table 1), galactose appears to give the best selectivity for the cancer cells, as no statistically significant interaction was detected with MCF-10A.

**Table 1:** Summary of statistically significant interactions (Welch two sample *t*-test) observed between PAA-glycan and each cell line^a^

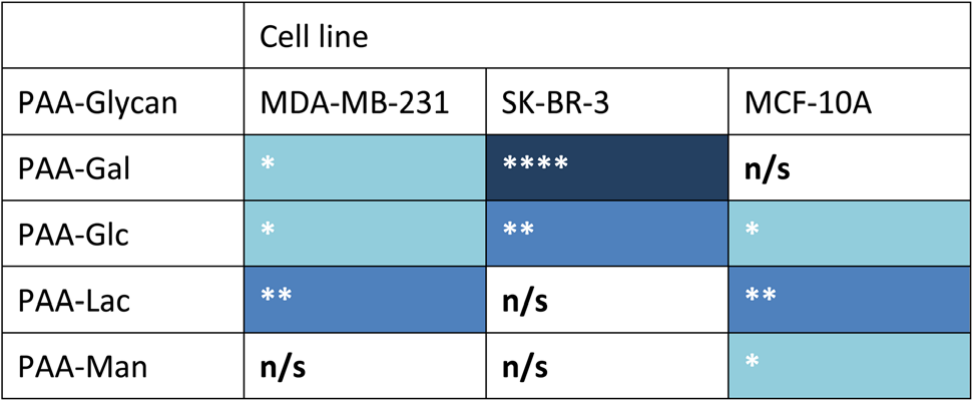

a Blue cell shading represents a statistically significant interaction observed between the PAA-glycan and the cell line: * = *p* < 0.05; ** = *p* < 0.01, and **** = *p* < 0.001 (dark blue being the strongest interaction observed); and white cells represents no statistically (n/s) significant interaction between the PAA-glycan and the cell line.

### 3.2 Glycan ligand synthesis

**Fig. 4:**
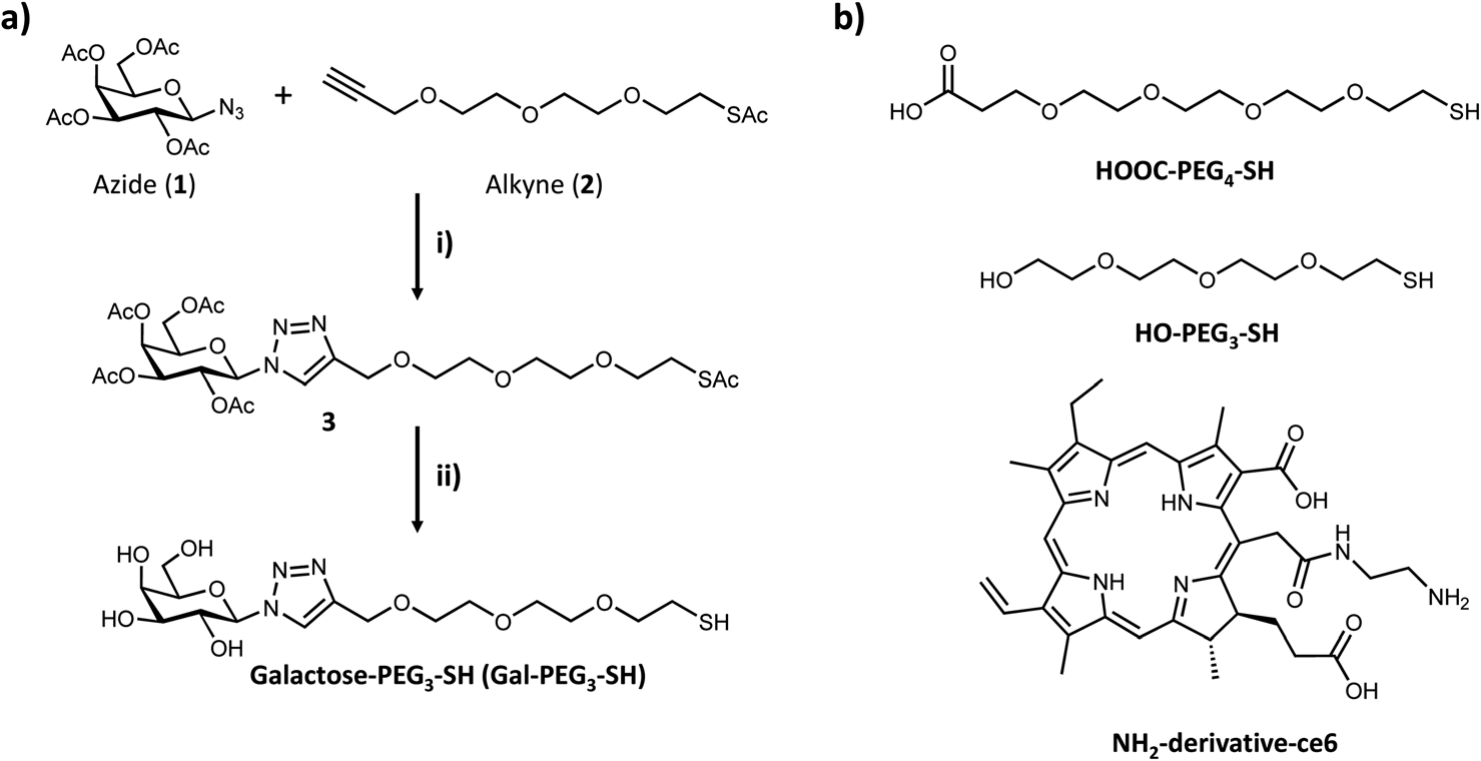
(a) Synthesis of Gal-PEG_3_-SH ligand: (i) azide (1), alkyne (2) 1.1 equiv., DMF, NaAsc 0.4 equiv., CuSO_4_ 0.2 equiv., THPTA 0.5 equiv., 50 °C, 16 h, yield 64%; and (ii) compound (3), dry MeOH, N_2_, NaMe O 5 equiv., r.t., 1 h, yield 87%. (b) Chemical structure of HOOC-PEG_4_-SH and HO-PEG_3_-SH ligands, and of the NH_2_-derivative-ce6 used as PS.

Based on the results obtained with the PAA-glycans, galactose proved to be the most promising glycan to target the cancer cells specifically and therefore the glycan that was moved forward for the construction of the nanoplatform to achieve targeted PDT of breast cancer cells. The three component nanosystem used herein consisted of 16 nm AuNPs containing the ce6 as the PS agent and galactose derivative ligand (Gal-PEG_3_-SH) to selectively direct the PDT reagent to the cancer cells surface. Gal-PEG_3_-SH was prepared through CuAAC chemistry as indicated in Fig. 4a. Azido (1) and alkyne (2) were prepared following known methodologies^52,53,62^ as indicated in the Materials and Methods section. Glycan-derivative 3 was obtained under standard CuAAC conditions,^63–67^ and deprotected by sodium methoxide yielding Gal-PEG_3_-SH (compound 4). Full characterisation of the compounds is reported in the Materials and methods section.

### 3.3 Synthesis and functionalisation of AuNPs

**Fig. 5:**
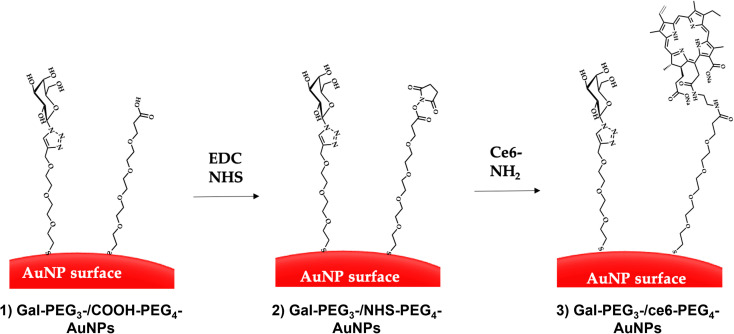
Direct conjugation of AuNPs through EDC/NHS coupling. The AuNPs were functionalised with glycan and carboxylated (PEG) ligands (step 1) through displacement of citrate ligands yielding glycan-PEG_3_-/COOH-AuNPs. EDC and NHS were added to the glycan-PEG_3_-/COOH-PEG_4_-AuNPs to generate NHS-activated particles (glycan-PEG_3_-/NHS-PEG_4_-AuNPs, step 2). Finally, the ce6-NH_2_ derivative is added to the particles to generate glycan-PEG_3_-/ce6-PEG_4_-AuNPs (step 3).

Citrate capped AuNPs (citrate-AuNPs, 16 nm) were prepared following the method developed by Turkevich *et al.*^54^ and subsequently used by our group,^22^ and yielded AuNPs with a surface plasmon absorption band centred at *ca.* 520 nm characteristic of *ca.* 16 nm AuNPs (Fig. S8†). The citrate-AuNPs were subjected to functionalisation with the different thiolated ligands (see Fig. 4 for detailed structures) for binding studies. To generate the Gal-PEG_3_-/ce6-PEG_4_-AuNPs (Fig. 5), a mixture of glycan ligand and carboxyl PEGylated thiol HOOC-PEG_4_-SH were added to the citrate-AuNPs, at a ratio of 3 : 1. The purified particles were then activated using EDC and NHS. An amine derivative of the ce6 (NH_2_-derivative-ce6, Fig. 4b) was then added to the activated particles, which generated the Gal-PEG_3_-/ce6-PEG_4_-AuNPs. A schematic representation of this synthesis is shown in Fig. 5. PEG_3_-/ce6-PEG_4_-AuNPs were synthesised as a control to evaluate the targeting capacity of the glycan and Gal-PEG_3_-AuNPs, without the photosensitiser ce6 were also synthesised as a control. The synthesised nanoparticles were characterised by UV-Vis absorption and MALDI-TOF (see Fig. S9–S12†).

For Gal-PEG_3_-/ce6-PEG_4_-AuNPs and PEG_3_-/ce6-PEG_4_-AuNPs (Fig. S9a and b,† respectively), the presence of the PS was confirmed by the absorption bands at *ca.* 400 nm and *ca.* 654 nm characteristic of Ce6.^68^ A red-shift in the extinction maximum was observed for all functionalised-AuNPs, suggesting successful functionalisation. The small changes in the FWHM and in the intensity at 800 nm following functionalisation confirm the absence of aggregation of the samples. When these nanoparticles were analysed by MALDI-TOF, the Gal-PEG_3_-SH and PEG_3_-SH ligands were detected in the positive mode, from the spectra in Fig. S10d and e,† respectively (highlighted with black arrows). However, the ce6-PEG_4_-SH was not detected in positive or negative mode (Fig. S10d and e†). Neither the ce6-NH_2_ ([ce6-NH_2_ + H]^+^ = 683 Da; [ce6-NH_2_ + Na]^+^ = 705 Da; [ce6-NH_2_ − H]^−^ = 681 Da) nor the ce6-PEG_4_-SH ([ce6-PEG_4_-SH + H]^+^ = 947 Da; [ce6-PEG_4_-SH + Na]^+^ = 969 Da; [ce6-PEG_4_-SH − H]^−^ = 945 Da) masses were detectable in the MALDI-TOF. Although there were broad signals around 683 Da, this signal was also detected in the citrate-AuNPs (Fig. S10b,† negative mode), which makes difficult to evaluate the presence of the ce6 signals. Consequently, it was not possible to use the MALDI-TOF to confirm ce6 conjugation. However, the presence of ce6 was detected by the characteristic signals in the UV-Vis spectra (Fig. S9†), and coupled with the MALDI-TOF data, the glycan and PEG ligands were detected in the functionalised suspensions. The presence of the corresponding ligand in Gal-PEG_3_-AuNPs was confirmed by the 2 nm red-shift compared to citrate-AuNPs observed in the UV-Vis extinction spectrum (Fig. S11†). The presence of the ligand on the Gal-PEG_3_-AuNPs was confirmed by MALDI-TOF (Fig. S12†), with the ligand detected in both the thiol – [Gal-PEG_3_-SH + Na]^+^ (432 Da), and disulfide [Gal-PEG_3_-S-S-PEG_3_-Gal + Na]^+^ (839 Da) – forms. The mass the Gal-PEG_3_-SH ligand, Fig. S12d† was detected in the purified Gal-PEG_3_-AuNPs suspension.

### 3.4 Singlet oxygen production by glycan-/ce6-PEG_4_-AuNPs

**Fig. 6:**
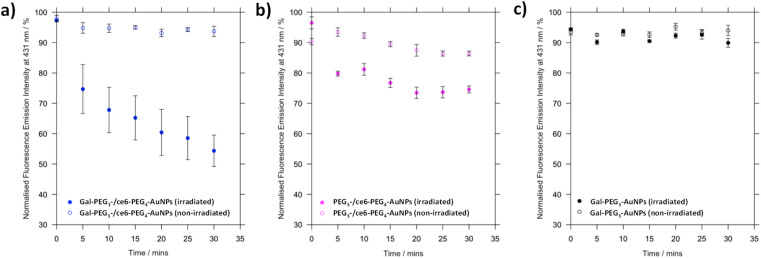
Processed fluorescence emission at 431 nm of ABMA, over 30 minutes in the presence of different functionalised AuNPs to monitor singlet oxygen generation by ce6. (a) Gal-PEG_3_-/ce6-PEG_4_-AuNPs, with (blue, filled circles) and without (blue circles) irradiation; (b) PEG_3_-/ce6-PEG_4_-AuNPs, with (magenta, filled circles) and without (magenta circles) irradiation; and (c) Gal-PEG_3_-AuNPs (control, no ce6) with (grey, filled circles) and without (grey circles) irradiation. Error bars = SE, *n* = 3. Samples were irradiated with a 633 nm He–Ne laser.

Before assessing cellular uptake of the synthesised nanoparticles and their potential for PDT of breast cancer cells, the ability of the ce6 installed on the nanoparticles to generate singlet oxygen was assessed (Fig. 6). For this, the fluorescent probe 9,10-anthracenediyl-bis(methylene)dimalonic acid (ABMA) was used, where the fluorescence of the probe was quenched on generation of singlet oxygen.^69^ A solution of Gal-PEG_3_-/ce6-PEG_4_-AuNP containing ABMA was irradiated with a 633 nm He–Ne laser, and the fluorescence measured every five minutes (Fig. 6a). To ensure the singlet oxygen generation was a result of irradiation with the 633 nm laser, the fluorescence emission of ABMA and Gal-PEG_3_-/ce6-PEG_4_-AuNPs was measured over time without irradiation. To confirm that any singlet oxygen generation was a result of the ce6 present on the AuNPs, Gal-PEG_3_-AuNPs (without ce6) were used as a control (Fig. 6c). Fig. 6 shows the normalised fluorescence emission intensity at 431 nm for each sample in the presence of ABMA, with (+) and without (−) irradiation. The percentage change in fluorescence after 30 minutes for each set of functionalised-AuNPs and ABMA solutions are shown in Table 2 (extracted from the data shown in Fig. 6). All ce6 containing particles showed a reduction in fluorescence intensity with irradiation indicating that singlet oxygen was generated. Gal-PEG_3_-/ce6-PEG_4_-AuNPs demonstrated the greatest quenching of the ABMA fluorescence signal, suggesting the greatest ability to generate singlet oxygen. From looking at the control AuNPs (Gal-PEG_3_-AuNPs), there was a background reduction of the fluorescence of ABMA by 4.5% with irradiation, where Gal-PEG_3_-/ce6-PEG_4_-AuNPs were below this background level without irradiation. Therefore, the Gal-PEG_3_-/ce6-PEG_4_-AuNPs were shown to generate singlet oxygen, in a light-dependent manner.

**Table 2:** Summary of the change in fluorescence at 431 nm for ABMA in the presence of Gal-PEG_3_-/ce6-PEG_4_-AuNPs, with and without irradiation. A negative value represents a decrease in fluorescence, and so quenching of the ABMA fluorescence from singlet oxygen generation. PEG_3_-/ce6-PEG_4_-AuNPs and **Gal-PEG**_**3**_**-AuNPs** were monitored as controls^a^

AuNPs	Change in fluorescence at 431 nm after 30 min/%	
	Irradiated	Not irradiated
Gal-PEG_3_/ce6-PEG_4_	−42.9	−4.3
PEG_3_-/ce6-PEG_4_	−21.9	−3.9
Gal-PEG_3_	−4.5	+0.6

a + = increase in fluorescence; − = decrease in fluorescence.

### 3.5 Glycan binding by cancer and non-cancer breast cell lines using glycans-/ce6-PEG_4_-AuNPs

**Fig. 7:**
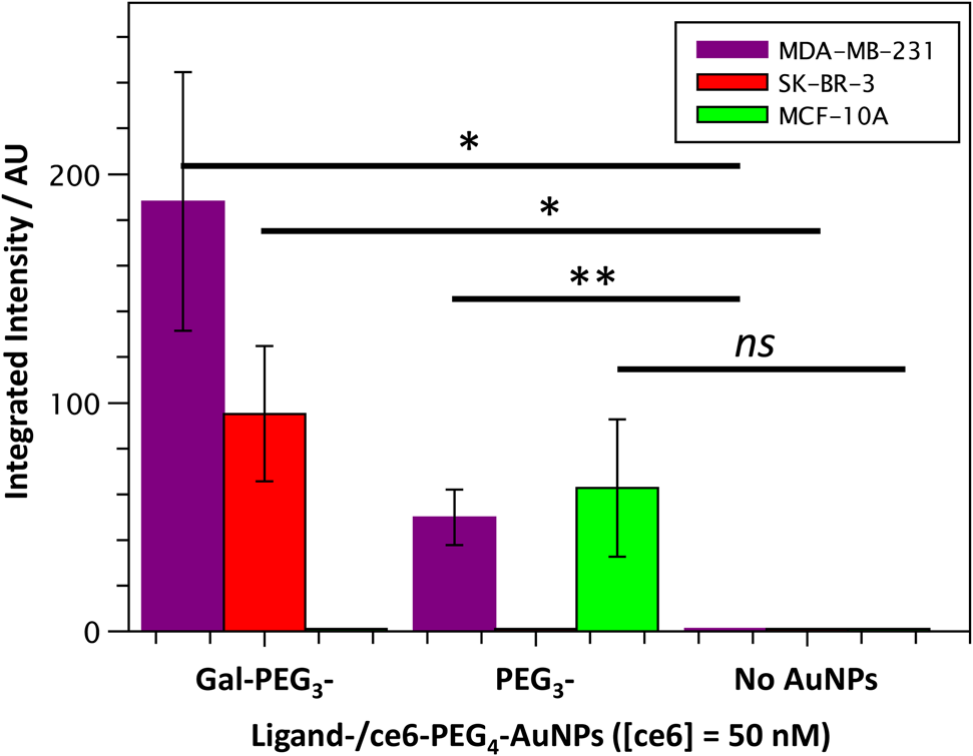
Quantitative analysis of confocal images from Gal-PEG_3_-/ce6-PEG_4_-AuNP uptake by MDA-MB-231 (purple), SK-BR-3 (red) or MCF-10A cells (green); showing the integrated intensity for the different AuNPs: Gal-PEG_3_-/ce6-PEG_4_-, PEG_3_-/ce6-PEG_4_- and no AuNPs, left to right. Error bars = +/− SEM, *n* = 7. * = *p* < 0.05; ** = *p* < 0.01; and ns = not significant (Welch two sample *t*-test, SEM = Standard Error of the Mean).

Once the Gal-PEG_3_-/ce6-PEG_4_-AuNPs were shown to be able to generate singlet oxygen following irradiation, they were assessed to evaluate their uptake by MDA-MB-231 and SK-BR-3 cancer cell lines. MC-10A were used as non-cancer cell line control and PEG_3_-/ce6-PEG_4_-AuNPs were used to assess any non-selective uptake by the cell lines. The cellular uptake of the AuNPs was monitored using confocal microscopy (Fig. S13–S15†) and the integrated intensity, from ce6, was measured to quantify the cellular uptake (Fig. 7). Confocal images were also used to investigate the localisation of the particles within the cells, using the BioTracker™ 560 Orange as lysosome dye. For the MDA-MB-231 cancer cell line, the localisation of the Gal-PEG_3_-/ce6-PEG_4_-AuNPs and PEG_3_-/ce6-PEG_4_-AuNPs was found mainly in acidic organelles but also elsewhere, suggesting that different receptors were involved for the uptake (Fig. S13†). When assessing the cell–glycan interaction, Gal-PEG_3_-/ce6-PEG_4_-AuNPs interacted with the MDA-MB-231 (Fig. 7, purple) demonstrating that the cell line is behaving as expected. In the SK-BR-3 cancer cell lines, the Gal-PEG_3_-/ce6-PEG_4_-AuNPs were the only particles tested that interacted with the cells, demonstrating a galactose selective uptake (Fig. 7, red). The localisation of the Gal-PEG_3_-/ce6-PEG_4_-AuNPs was predominantly not within in acidic organelles (Fig. S14†). Previous studies by our group^31^ indicated targeting of SK-BR-3 when using lactose-PS functionalised AuNPs, which supports the strong cellular uptake observed with Gal-PEG_3_-/ce6-PEG_4_-AuNP. The results from the AuNP uptake by SK-BR-3 matched the results from the PAA-glycan studies, as PAA-Gal had the highest level of uptake by SK-BR-3 (Fig. S5†). Control experiments (Fig. 7 – green and Fig. S15†) showed that no cellular uptake or interaction was observed between the Gal-PEG_3_-/ce6-PEG_4_-AuNPs and the non-cancer MCF-10A cell line. The PEG_3_-/ce6-PEG_4_-AuNPs had mixed results, and showed low uptake that was variable between images, where some of the images had no uptake at all, suggesting that the interaction was non-selective and was determined as not statistically significant (using a Welch two sample *t*-test).

### 3.6 Receptor uptake of Gal-PEG_3_-/ce6-PEG_4_-AuNPs by breast cancer cell lines

As the experiments described so far strongly suggest that the same glycan-AuNPs system might interact with different receptors of each cell line (galectin or GLUT receptors for MDA-MB-231 and SK-BR-3, respectively), the interaction of the cancer cell lines with Gal-PEG_3_-/ce6-PEG_4_-AuNPs in the presence of different galactose-binding protein inhibitors was tested. The inhibitors investigated (Table S2†) included inhibitors of: GLUTs (WZB117), galectins (33DFTG), and SGLTs (canagliflozin). The cancer cell lines were incubated with 50 μM of each inhibitor for one hour before addition of the particles and imaged by confocal microscopy. Confocal images of Gal-PEG_3_-/ce6-PEG_4_-AuNPs uptake by both cancer cell lines and in the presence (or absence) of the different galactose-binding protein inhibitors are reported in Fig. S16† (MDA-MB-231 cell line) and Fig. S17† (SK-BR-3 cell line). In MDA-MB-231 cells, Gal-PEG_3_-/ce6-PEG_4_-AuNPs interaction was inhibited in the presence of the galectin inhibitor (33DFTG) to high significance (*p* < 0.005, Fig. S18a†). The SGLT and GLUT inhibitors did not show any significant inhibition for Gal-PEG_3_-/ce6-PEG_4_-AuNPs interaction with the MDA-MB-231 cell line. Although there appeared to be a reduction in mean integrated intensity upon addition of WZB117, compared to no inhibitor, the reduction was not statistically significant (*p* = 0.1229). In SK-BR-3, the GLUT receptor inhibitor (WZB117) showed significant inhibition of the Gal-PEG_3_-/ce6-PEG_4_-AuNPs interaction with this cancer cell line (Fig. S18b†); and there was no significant inhibition with the SGLT or galectin inhibitors. The results demonstrated that the cell lines used different proteins for Gal-PEG_3_-/ce6-PEG_4_-AuNPs uptake. For MDA-MB-231 (high expression of galectin-1), galectin inhibitor significantly reduced NP uptake by the cell line. Whereas for SK-BR-3, the GLUT inhibitor significantly reduced NP uptake. Consequently, cellular uptake of the Gal-PEG_3_-/ce6-PEG_4_-AuNPs by the cancer cell lines were both galactose–selective interactions.

### 3.7 Targeted PDT against breast cancer cell lines using Gal-PEG_3_-/ce6-PEG_4_-AuNPs, *in vitro*

**Fig. 8:**
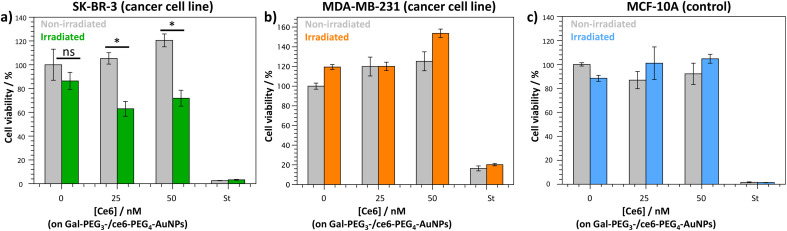
CellTitre Blue cell viability results after PDT treatment using Gal-PEG_3_-/ce6-PEG_4_-AuNPs against (a) SK-BR-3 (breast cancer cell line); (b) MDAMB-231 (breast cancer cell line); and (c) MCF-10A (control cells). Different concentrations of Gal-PEG_3_-/ce6-PEG_4_-AuNPs were tested (0, 25 and 50 nM, left to right), with 20 μM staurosporine (st) as a positive control for cell death. Error bars = ±SEM, *n* = 3. * = *p* < 0.05 and ns = not significant (Welch two sample *t*-test, SEM = Standard Error of the Mean).

The Gal-PEG_3_-/ce6-PEG_4_-AuNPs were taken forward for PDT studies against the breast cancer cell lines MDA-MB-231 and SK-BR-3 using a CellTitre Blue cell viability assay (Fig. 8). The non-cancer breast cell line MCF-10A was used as a control cell line to assess selective toxicity of the particles. For the PDT studies, 20 μM of staurosporine (st) was used as a positive control, as it is toxic to cells at this concentration. Different concentrations of the Gal-PEG_3_-/ce6-PEG_4_-AuNPs were used, based on the ce6 concentration of the particle solution (0, 25 or 50 nM). For each set of conditions, the studies were performed in duplicates to have one set of conditions exposed to irradiation (irradiated), and a corresponding control set of conditions that were not exposed to any light (non-irradiated). The data in Fig. 8 is represented as a percentage of cell viability, normalised to the non-irradiated, non-treated cells (0 nM of particles). The Gal-PEG_3_-/ce6-PEG_4_-AuNPs successfully killed SK-BR-3 cell line upon irradiation with the laser using 50 nM and 25 nM ce6, with a 48% and 42% reduction in cell viability, respectively compared to the control cells (green bars). A 13% reduction in cell viability was observed for untreated SK-BR-3 irradiated under the same conditions. However, there was no detectable cell killing of the MDA-MB-231 cell line at the same concentration. No cell death of the non-cancer breast cell line (MCF-10A) was detected at 50 nM, demonstrating selective killing of the SK-BR-3 cells by 50 nM Gal-PEG_3_-/ce6-PEG_4_-AuNPs. The reasons behind the lack of cell death in MDA-MB-231 samples are not clear, as the confocal images showed interaction between the Gal-PEG_3_-/ce6-PEG_4_-AuNPs and the MDA-MB-231 cell line. One possibility to rationalise the results was the localisation of the particles in the MDA-MB-231 cells. As seen in the confocal work, the Gal-PEG_3_-/ce6-PEG_4_-AuNPs reside predominantly in the acidic organelles of MDA-MB-231 cells, whereas this was not the case for the SK-BR-3 cell line. Even though both cells appear to take up the Gal-PEG_3_-/ce6-PEG_4_-AuNPs, the localisation of the photosensitiser has been shown important for the efficiency of cell death. As reactive oxygen species have a short life-time, their location can be critical for achieving effective cell death. Although localisation in acidic organelles such as lysosomes has been shown effective for photoinduced cell death, organelles such as the mitochondria are widely superior for achieving efficient cell death.^70,71^ One thought for the reason behind this improved efficiency is that the mitochondria directly induces apoptotic cell death, whereas lysosomes relies on indirect methods.^71^

## 4. Conclusions

The research carried out here determined a suitable glycan to target breast cancer cells for PDT. Through confocal microscopy, two different scaffolds were employed to probe glycan binding and subsequent uptake by the cancer cell lines: MDA-MB-231 and SK-BR-3; and then compare their glycan binding to a non-cancer breast epithelial cell line: MCF-10A. First, a selection of PAA-glycans were chosen to assess glycan uptake by the cell lines. The PAA-glycans were commercially available and so were easily accessible to test an array of different glycans, including galactose, glucose, lactose, and mannose. From the PAA-glycan analysis, the different cell lines showed different selectivity and uptake strength towards each PAA-glycan. However, PAA-Gal demonstrated a selective interaction with both cancer cell lines, with no significant uptake by the non-cancer cell line. With this information, a novel galactose derivative ligand, Gal-PEG_3_-SH (4) was synthesised *via* CuAAC chemistry where the glycans were ‘clicked’ to an alkyne PEGylated thiol derivative. The glycan ligand was installed on *ca.* 16 nm AuNPs as carriers, which were further decorated with a ce6-PEG_4_-SH derivative as the PS drug. Gal-PEG_3_-/ce6-PEG_4_-AuNPs were able to produce singlet oxygen upon irradiation with a 633 nm laser and the role of the PS in this production was confirmed by the non-production of singlet oxygen by the control Gal-PEG_3_-AuNPs. Consistent with the PAA-glycan analysis, Gal-PEG_3_-/ce6-PEG_4_-AuNPs showed selective interactions with the cancer cell lines, with no significant interaction detected with the non-cancer cell line at 50 nM. Interestingly, there was a difference between the proteins involved in cellular uptake of the particles between the two cancer cell lines. Gal-PEG_3_-/ce6-PEG_4_-AuNPs interaction with MDA-MB-231 was inhibited by 33DFTG, an inhibitor of galectin-1 and -3, demonstrating that galectins were involved in cellular uptake of the nanoparticles by MDA-MB-231 cells. For SK-BR-3, Gal-PEG_3_-/ce6-PEG_4_-AuNPs was inhibited by WZB117, an inhibitor of GLUT-1, -3 and -4, and therefore particle uptake was regulated by GLUT receptors on SK-BR-3. The SGLT inhibitor did not affect the Gal-PEG_3_-/ce6-PEG_4_-AuNPs interaction with either cell line, showing that SGLT does not play a major role in particle uptake. The Gal-PEG_3_-/ce6-PEG_4_-AuNPs were able to induce targeted cell death of breast cancer cells lines through PDT, with a 46.3% reduction in cell viability upon irradiation observed in SK-BR-3 cells treated with 50 nM ce6. No detectable cell killing was observed for the control cell line MCF-10A, demonstrating selective cell killing of the SK-BR-3 cell line with Gal-PEG_3_-/ce6-PEG_4_-AuNPs. However, the MDA-MB-231 cell line did not show cell death with 50 nM ce6, even though particle uptake at this concentration was demonstrated in the confocal microscopy studies.

Further investigations will involve determining the localisation for the PS ce6 in the SK-BR-3 cells to support optimisation of the conditions for the MDA-MB-231 cell killing. This could be achieved by altering the incubation time and changing the concentrations, as both can affect localisation of the PS in the cells. For example, if the PS localises in the plasma membrane, low concentrations can induce cell proliferation, and higher doses would be required to induce damage to the plasma membrane.^72^ However, high concentrations of PS in lysosomes can reduce the efficiency of photoinduced cell death, as they can form aggregates.^73^ What was clear from the microscopy results was that the two cancer cell lines rely on different proteins for NP uptake. Consequently, this could mean different rates of uptake, or cellular processing once taken up by the cells, both of which could impact cell eradication as localisation of the PS is key to effective PDT treatment. Further research will also evaluate the sequestration of the glycoAuNPs by macrophages as recent reports have indicated how the morphology of glyconanoparticles and other glycomaterials can impact macrophage cellular uptake and immune response.^74–76^

The findings reported herein demonstrate the versatility of glycoAuNPs for selective binding to a cellular target, through glycan–lectin interactions. Further, the selective cell killing of breast cancer cells demonstrates the potential of using this approach for targeted PDT.

## Conflicts of interest

The authors declare that they have no known competing financial interests or personal relationships that could have appeared to influence the work reported in this paper.

## Supplementary Material

NA-005-D3NA00544E-s001
