# Supplementary material for: Targeted photodynamic therapy for breast cancer: the potential of glyconanoparticles
Source: Nanoscale Adv. 2023 Oct 27;5(23):6501–13. doi: 10.1039/d3na00544e (PMC10662151; doi:10.1039/d3na00544e)
Supplement: NA-005-D3NA00544E-s001 [file NA-005-D3NA00544E-s001.pdf]

Electronic Supplementary Material (ESI) for Nanoscale Advances.
This journal is © The Royal Society of Chemistry 2023

SUPPORTING INFORMATION

Targeted photodynamic therapy for breast cancer: the potential of glyconanoparticles

Brydie A. Thomas-Moore,[a,b] Simone Dedola,[a,c] David A. Russell,[b] Robert A. Field[a,c] and María J. Marín,[b*]

[a]Iceni Glycoscience Ltd., Norwich Research Park, Norwich, NR4 7TJ, UK

[b]School of Chemistry, University of East Anglia, Norwich Research Park, Norwich, NR4 7TJ, UK

[c]Department of Chemistry and Manchester Institute of Biotechnology, University of Manchester, 131 Princess Street, Manchester, M1 7DN, UK

*Correspondence: m.marin-altaba@uea.ac.uk

Index

1. Materials and methods

1.1 General……………………………………………………………………………………3

1.2 Instrumental techniques……………………………………………………………………3

1.2.1 Mass spectrometry……………………………………………………………………3

1.2.2 Nuclear magnetic spectroscopy………………………………………………………3

1.2.3 UV-Vis spectroscopy…………………………………………………………………3

1.2.4 Fluorimetry……………………………………………………………………………4

1.2.5 Confocal laser scanning microscopy and image analysis……………………………4

1.3 Buffers and media…………………………………………………………………………5

1.4 Annotations for NMR assignment of glycan ligands………………………………………5

1.5 Cell culturing………………………………………………………………………………6

1.5.1 Human cell lines culture………………………………………………………………6

1.5.2 Starting new cell cultures……………………………………………………………6

1.5.3 Subculturing…………………………………………………………………………6

2. Results

2.1 Assessing glycan uptake using glycan functionalised polyacrylamide polymers……………7

2.1.1 Cancer cell line: MDA-MB-231…………………………………………………………7

2.1.2 Cancer cell line: SK-BR-3………………………………………………………………9

2.1.3 Healthy cell line: MCF-10A……………………………………………………………11

2.2 Synthesis of citrate-AuNPs…………………………………………………………………12

2.3 Functionalisation of citrate-AuNPs…………………………………………………………13

2.3.1 Gal-PEG$_3$-/ce6-PEG$_4$-AuNPs and PEG$_3$-/ce6-PEG$_4$-AuNPs……………………………13

2.3.2 Gal-PEG$_3$-AuNPs………………………………………………………………………15

2.4 Glycan uptake by cancer and non-cancer breast cell lines using glycan-/ce6-PEG$_4$-AuNPs………16

2.4.1 Cancer cell line: MDA-MB-231…………………………………………………………16

2.4.2 Cancer cell line: SK-BR-3………………………………………………………………17

2.4.3 Healthy cell line: MCF-10A……………………………………………………………18

2.5 Receptor uptake of Gal-PEG$_3$-/ce6-PEG$_4$-AuNPs by breast cancer cell lines………………18

1. Materials and Methods

1.1 General

All reagents were of analytical grade and purchased from Sigma Aldrich, Merck, Fischer Scientific or Thermo Fischer Scientific, unless stated otherwise. PAA-glycans (with molecular weight ca. 2,800 g/mol) were purchased from GlycoTech. The SK-BR-3 cell line was kindly provided by Professor Dylan Edwards (University of East Anglia, UK), which was obtained from LGC Standards. MDA-MB-231 and MCF-10A were purchased from ATCC (LGC Standards).

1.2 Instrumental techniques

1.2.1 Mass spectrometry

Two mass spectrometry methods were used. Firstly, ESI-MS was used during ligand synthesis (Advion Expression Compact Mass Spectrometer). Through direct injection, 10 µL of *ca.* 0.1 mg·mL^{-1} was injected. The masses were recorded in positive mode and analysed using Advion Mass Express software. Secondly, MALDI-TOF MS analysis was used (Bruker Daltonics autoflex speed ToF/ToF mass spectrometer). Concentrated samples (*ca.* 150 nM AuNPs) were prepared by performing a 1: 1 dilution of sample in DHB matrix, and then 2 µL of the mixture was spotted onto a MTP AnchorChip 384 target plate. The spot was left to dry at room temperature before analysis. The MALDI-TOF MS equipment used a nitrogen laser, and analysis was performed in a linear 50 shot mode, with 32x gain and 70% laser intensity.

1.2.2 Nuclear magnetic resonance spectroscopy

Nuclear magnetic resonance (NRM) was performed in a Bruker Avange III HD 400 MHz. The instrument used a broadband BBFO probe at 400 MHz (1H) and 100 MHz (13C) at 298 °C. For the analysis, the chemical shifts (δ) are in parts per million (ppm). Compounds were assigned using proton, carbon, HSQC edited two-dimensional correlation spectroscopy and COSY. Assignment was performed using Mestrenova software (Mestrelab Research, S.L.).

1.2.3 UV-Vis spectroscopy

UV-Vis measurements were obtained using a Varian Cary® 50 UV-Vis spectrophotometer or a BMG Labtech CLARIOstar® high-performance microplate reader. For the UV-Vis spectrophotometer measurements, samples (1 mL) were added to a quartz cuvette with a 1 cm path length and measured using Cary WinUV software. For the plate reader measurements, samples (50 µL) were loaded into 384-well microtitre plates (4titude), and measurements recorded using Omega series and MARS Data Analysis software (BMG Labtech).

1.2.4 Fluorimetry

Fluorescence measurements were obtained using a Hitachi F-4500 fluorimeter or a BMG Labtech CLARIOstar® high-performance microplate reader. For the fluorimeter measurements, samples (1 mL) were added to a Quartz cuvette with a 1 cm path length, and measurements were recorded using FluorEssence™ (Horiba) software. For the plate reader measurements, samples (120 µL) were loaded into Nunc Nunclon™ Surface 96-well microtitre plates, and measurement were recorded using Omega series and MARS Data Analysis software (BMG Labtech).

1.2.5 Confocal laser scanning microscopy and image analysis

A Carl Zeiss LSM 510 META confocal laser scanning microscope with a plan-apochromat 63x/1.4 Oil DIC objective was used to obtain the images, and the data was processed using ImageJ/Fiji software. For quantification of fluorescence, each image was despeckled, followed by setting the minimum threshold to 23, with the 'Triangle' threshold method. The threshold was determined using the condition which provided the highest grey value, *i.e.*, the strongest interaction. The threshold was determined at the point where all background signal was removed and only fluorescence due to the dye interaction with the cell was measured. The threshold was then applied throughout all images for analysis. Fluorescence within the cells was represented and quantified by the integrated intensity, which was determined as follows. The integrated intensity of one image is derived from the following equation (Eq. 1):

$$I = \frac{(Mean\ Grey\ Value\ \times\ Area)}{n} \qquad \text{(Eq. 1)}$$

Where the mean grey value is the average intensity of all pixels measured. The area is the total area of the selection within the image where the grey values were recorded, and *n* is the number of cells within that image.

The mean integrated intensity is consequently calculated using Eq. 2:

$$Mean\ I = \frac{\sum I}{n} \qquad \text{(Eq. 2)}$$

Where $\sum I$ is the sum of all integrated intensity for all images within one condition, and *n* is the number of images analysed using that condition.

1.3 Buffers and media

The composition of the buffers and media supplements used in this research is summarised in **Table S1**. The pH of each buffer was corrected with 1 M NaOH or 1 M HCl. All buffers and media that were used for cell culture were autoclaved at 110 °C for 10 minutes, and sterilised using a 0.22 µm syringe filter (Millex GP).

Table S1: List of buffers and their composition.

Buffer/Media	Composition
**PB**	10 mM Phosphate buffer
**PBS**	10 mM PB with 150 mM NaCl, pH 7.4
**MES**	10 mM MES buffer, pH 5.5
**Imaging medium**	NaCl (120 mM), KCl (5 mM), $CaCl_2$ (2 mM), $MgCl_2$ (1 mM), monosodium phosphate (1 mM), sodium bicarbonate (1 mM), HEPES (25 mM), D-glucose (11 mM) and BSA (1 mg·mL^{-1}) in 50 mL of PB, pH 7.4
**DMEM(-)**	DMEM with 1% penicillin/streptomycin solution and phenol red
**DMEM(+)**	DMEM with 1% penicillin/streptomycin solution, phenol red and 10% FBS
**Freezing medium**	DMEM(+) or MEGM, with 10% DMSO
**MEGM**	MEBM™ Basal Medium with MEGM™ Bullet Kit (BPE (2 mL), hydrocortisone (0.5 mL), hEGF (0.5 mL) and insulin (0.5 mL))

1.4 Annotations for NMR assignment of glycan ligands

a)

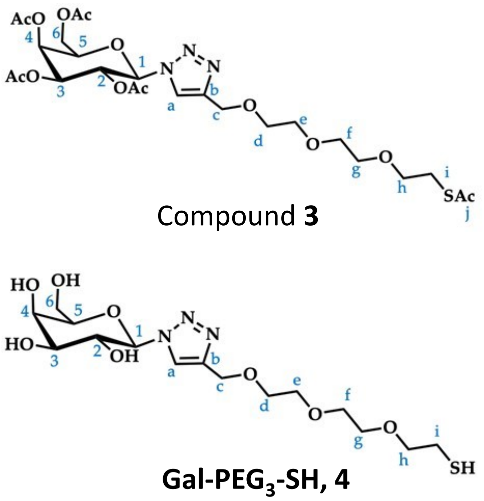

Compound **3**

b)

Gal-PEG$_3$-SH, 4

Figure S1. a) Structure of compound **3**, S-10-(1(2,3,4,6-Tetra-O-acetyl-β-D-galactopyranosyl)-1H-1,2,3-triazol-4yl)-3,6,9-trioxadecyl ethanethioate (Ac-galactose-PEG$_3$-SAc), with annotations for NMR assignment. **b)** Structure of 10-(1-(β-D-galactopyranosyl)-1H-1,2,3-triazol-4yl)-3,6,9-trioxadecylthiol (**Gal-PEG$_3$-SH**) with annotations for NMR assignment.

1.5 Cell culturing

1.5.1 Human cell lines culture

All cell culture was performed under sterile conditions. The cancer cell lines, MDA-MB-231 and SK-BR-3, were cultured in DMEM(+), and the MCF-10A cell line was cultured in MEGM. All reagents were heated to 37 °C before addition to the cells.

1.5.2 Starting new cell cultures

Vials of the three cell lines were removed from storage in liquid nitrogen. The cells were thawed at room temperature. To the thawed cells, 1 mL of media was added, and using a pipette, the cells were gently mixed to ensure dispersion into the media. The cells were then transferred to 75 cm^2 Nunc Easy flasks, and a further 10 mL of media was added to make a total volume of 12 mL. All the flasks were then incubated at 37 °C, 5% CO_2 atmosphere. After 24 hours, the media was removed and replenished with 12 mL of fresh media to remove any residual DMSO from the freezing medium.

1.5.3 Subculturing

The cells were subcultured every five days. The cell culture media was discarded and washed with 5 mL of PBS. Next, 5 mL of 0.25% trypsin EDTA was added to the flasks and incubated at 37 °C for 5 mins. The flasks were tapped, to dislodge the cells. To quench the trypsin, 5 mL of DMEM(+) was added to the flasks containing the cancer cell lines. For the MCF-10A cell line, 5 mL of soybean trypsin inhibitor solution (1 $mg{\cdot}mL^{-1}$, in PBS) was added to quench the trypsin. The solutions were added to a 15 mL falcon tube and centrifuged at 800 rcf for 5 mins (MDA-MB-231 and SK-BR-3), or 130 rcf for 7 mins (MCF-10A). The pellets were resuspended in 10 mL of media. The cultures were then diluted 1: 4 in media, transferred into fresh culture flasks, and incubated at 37 °C at 5% CO_2.

2. Results

2.1 Assessing glycan uptake using glycan functionalised polyacrylamide polymers

2.1.1 Cancer cell line: MDA-MB-231

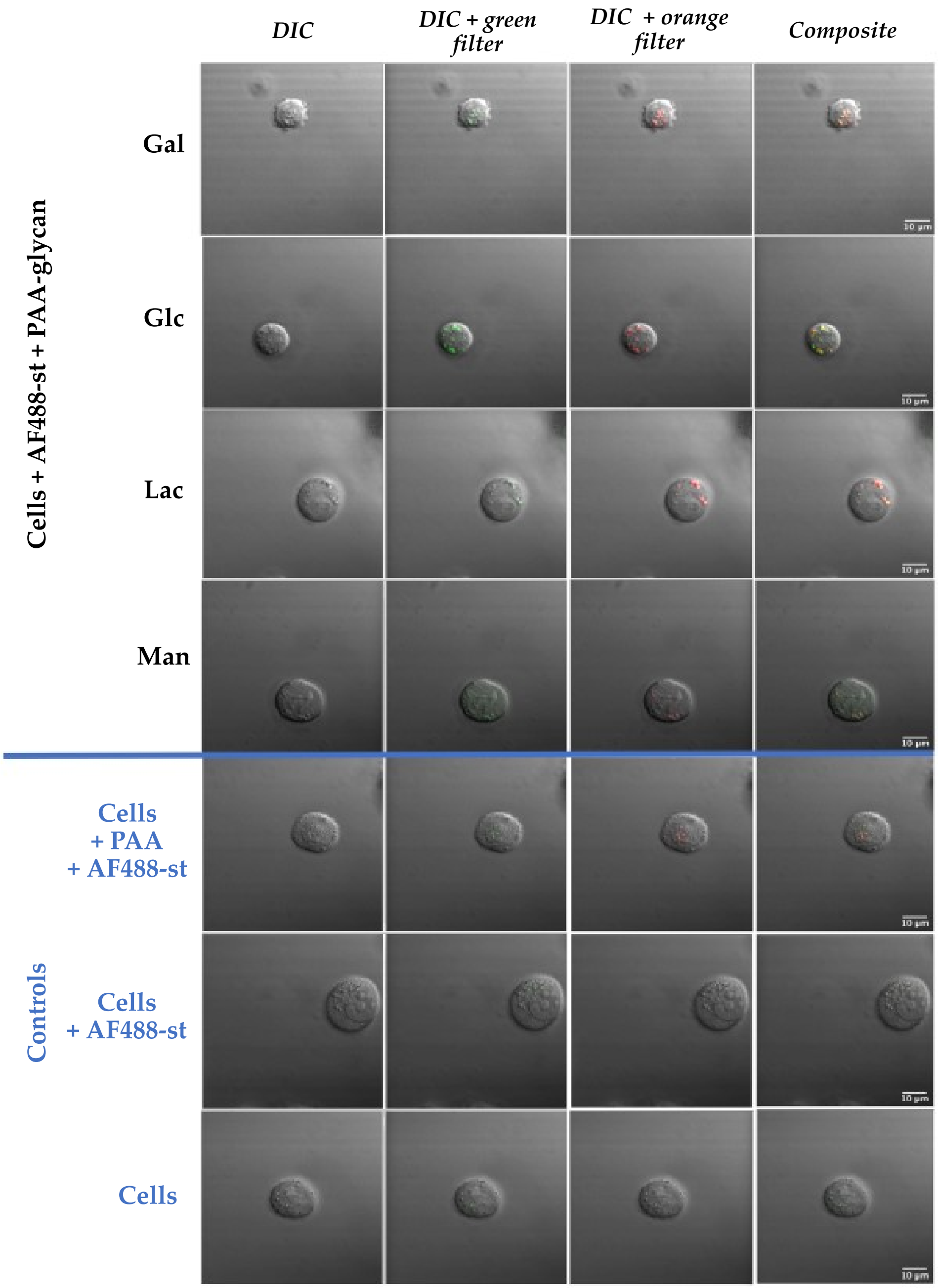

Figure S2. Example of confocal images from PAA-glycan (galactose (Gal), glucose (Glc), lactose (Lac) and mannose (Man), top to bottom) uptake by MDA-MB-231. Images are separated into differential interference contrast image (DIC), AF488-st (green filter, λ_{exc} = 488 nm, $\Delta\lambda_{em}$ = 505 – 530 nm), BioTracker Orange dye (orange filter, λ_{exc} = 514 nm, $\Delta\lambda_{em}$ = >550 nm) and composite, from left to right. AF488-st represents glycan uptake, and BioTracker Orange dye represents acidic organelles.

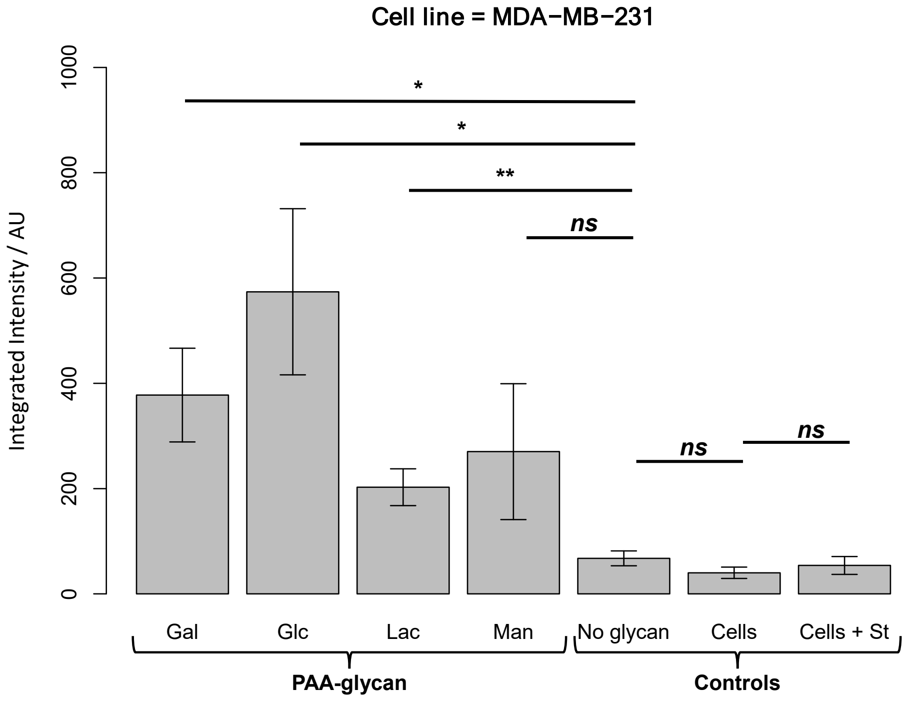

Figure S3. Quantitative analysis of confocal images from PAA-glycan uptake by MDA-MB-231, showing the integrated intensity for each glycan (galactose, glucose, lactose and mannose, left to right) and controls. Error bars = ± SEM, n = 7. * = $p < 0.05$; ** = $p < 0.01$; and ns = not significant ($p > 0.05$) (Welch two sample t-test).

2.1.2 Cancer cell line: SK-BR-3

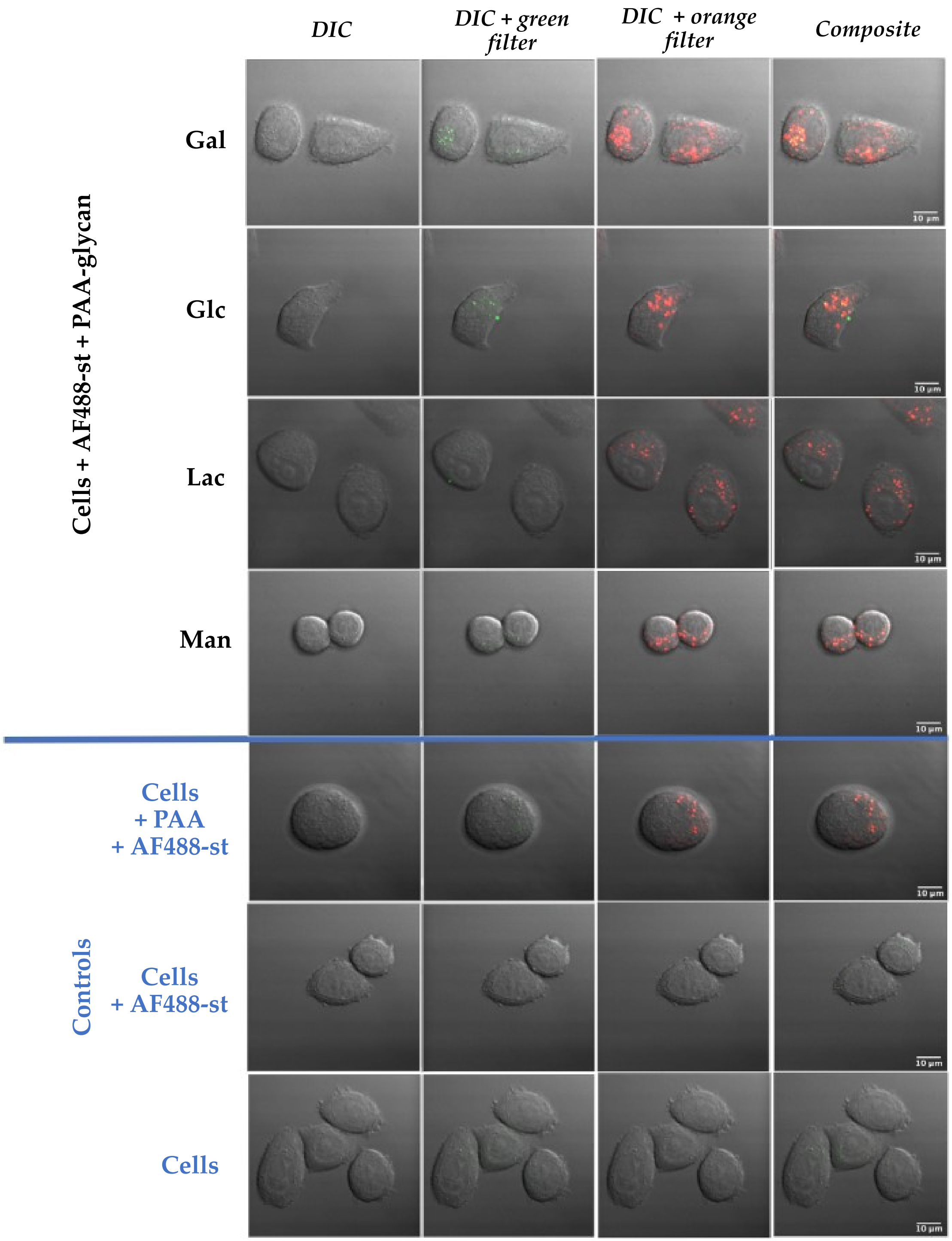

Figure S4. Example of confocal images from PAA-glycan (galactose, glucose, lactose and mannose, top to bottom) uptake by SK-BR-3. Images are separated into no filter (DIC image), AF488-st (green filter, λ_{exc} = 488 nm, $\Delta\lambda_{em}$ = 505 – 530 nm), BioTracker Orange dye (orange filter, λ_{exc} = 514 nm, $\Delta\lambda_{em}$ = >550 nm) and composite, from left to right. AF488-st represents glycan uptake, and BioTracker Orange dye represents acidic organelles.

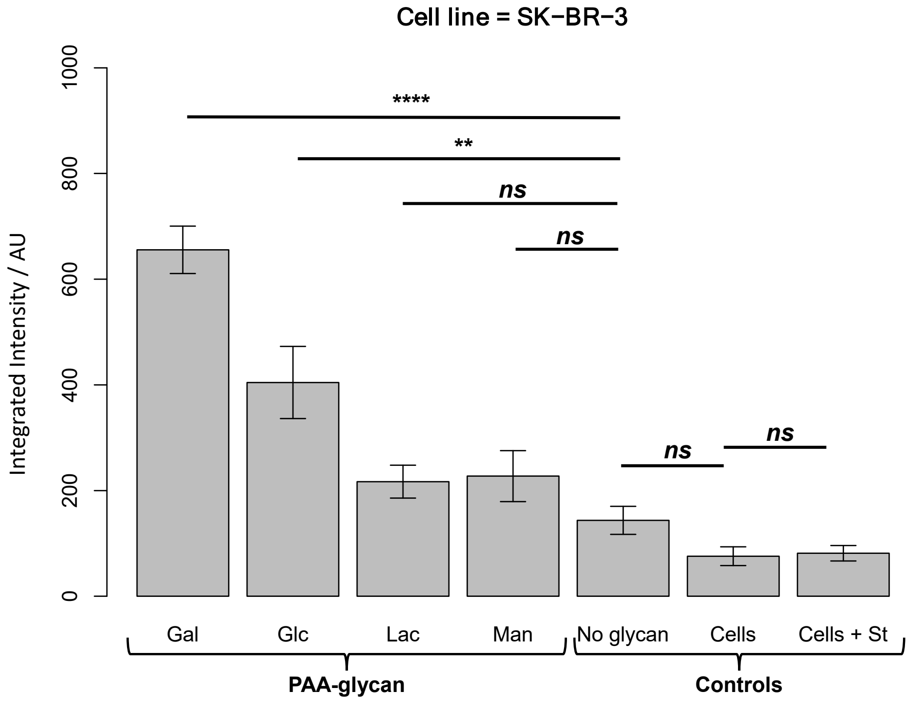

Figure S5. Quantitative analysis of confocal images from PAA-glycan uptake by SK-BR-3 cells, showing the integrated intensity for each glycan (galactose, glucose, lactose and mannose, left to right) and controls. Error bars = +/- SEM, n=7. ** = $p < 0.01$; **** = $p < 0.001$; and ns = not significant ($p > 0.05$) (Welch two sample t-test).

2.1.3 Healthy cell line: MCF-10A

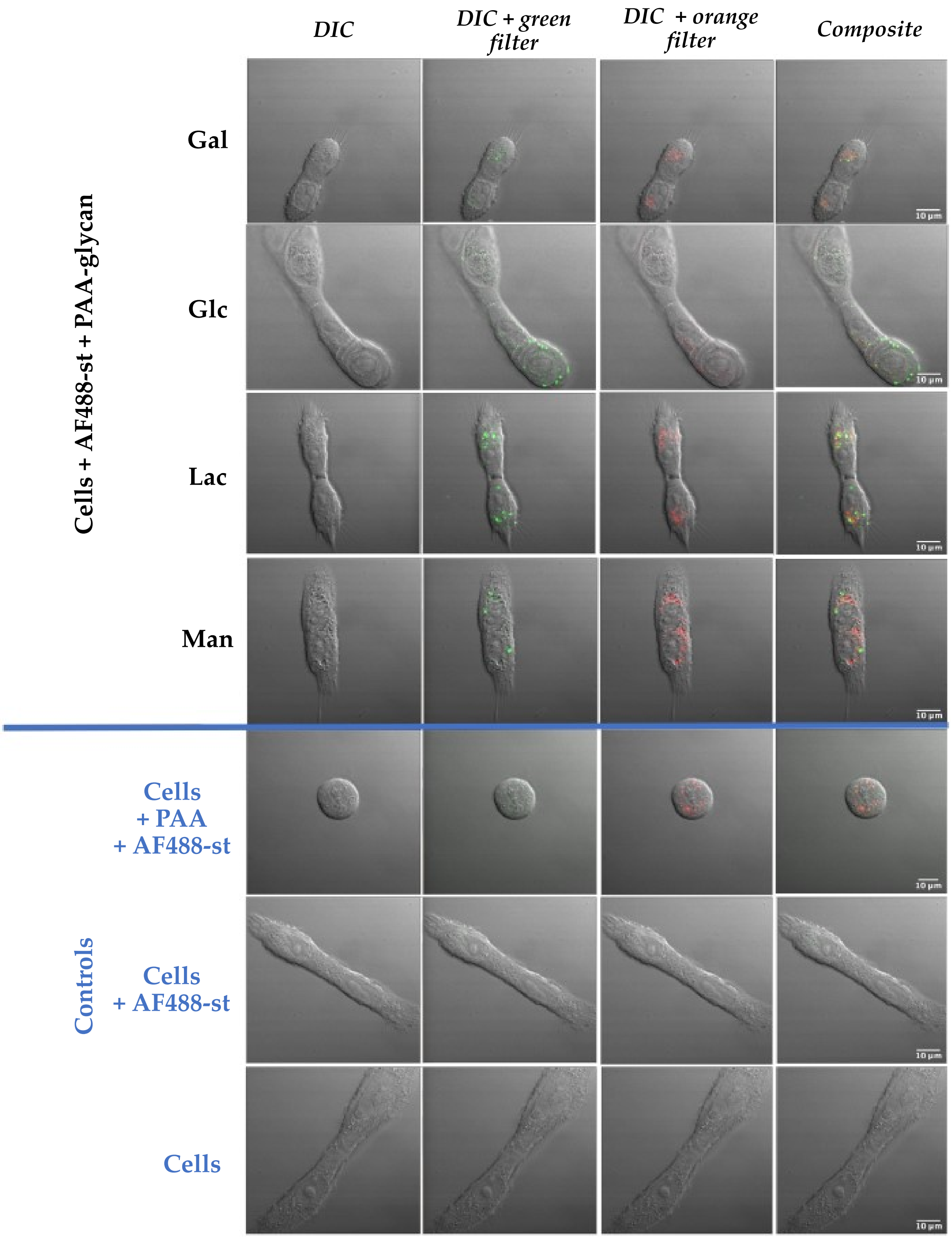

Figure S6. Example of processed confocal images from PAA-glycan (galactose, glucose, lactose and mannose, top to bottom) uptake by MCF-10A. Images are separated into no filter (DIC image), AF488-st (green filter, λ_{exc} = 488 nm, $\Delta\lambda_{em}$ = 505 – 530 nm), BioTracker Orange dye (orange filter, λ_{exc} = 514 nm, $\Delta\lambda_{em}$ = >550 nm) and composite, from left to right. AF488-st represents glycan uptake, and BioTracker Orange dye represents acidic organelles.

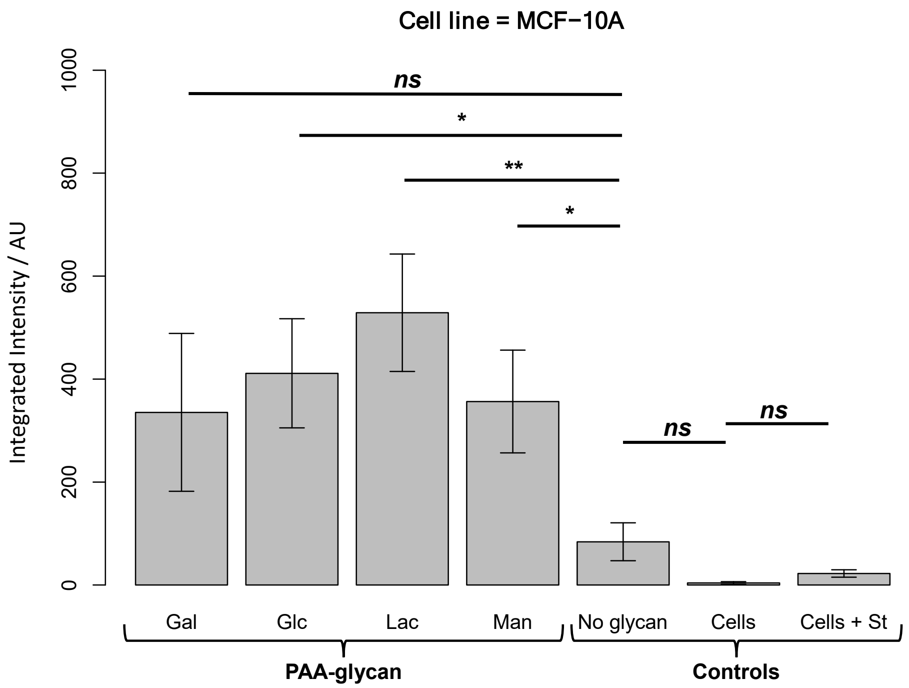

Figure S7. Quantitative analysis of confocal images from PAA-glycan uptake by MCF-10A cells, showing the integrated intensity for each glycan (galactose, glucose, lactose and mannose, left to right) and controls. Error bars = +/- SEM, n=7. * = $p < 0.05$; ** = $p < 0.01$; and ns = not significant ($p > 0.05$) (Welch two sample t-test).

2.2 Synthesis of citrate-AuNPs

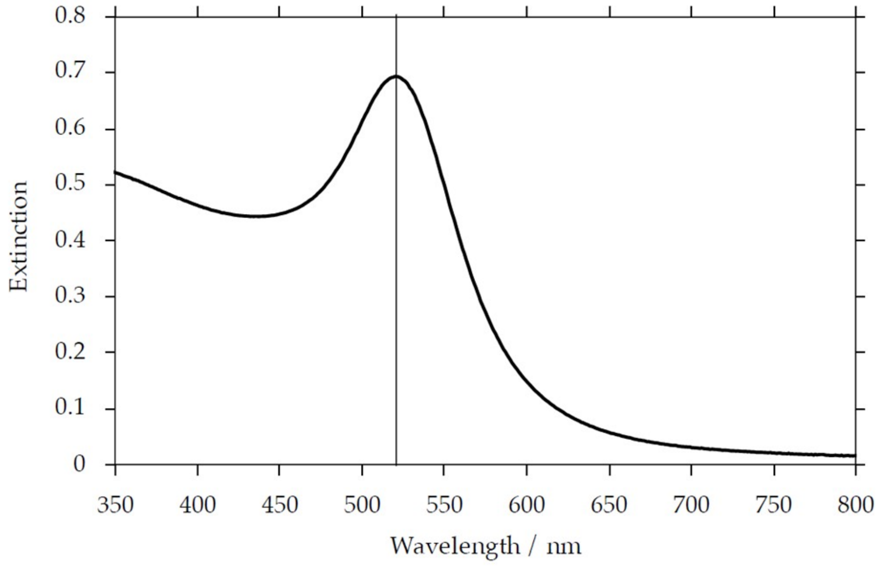

Figure S8. UV-Vis extinction spectrum of citrate-AuNPs.

2.3 Functionalisation of citrate-AuNPs

2.3.1 Gal-PEG$_3$-/ce6-PEG$_4$-AuNPs and PEG$_3$-/ce6-PEG$_4$-AuNPs

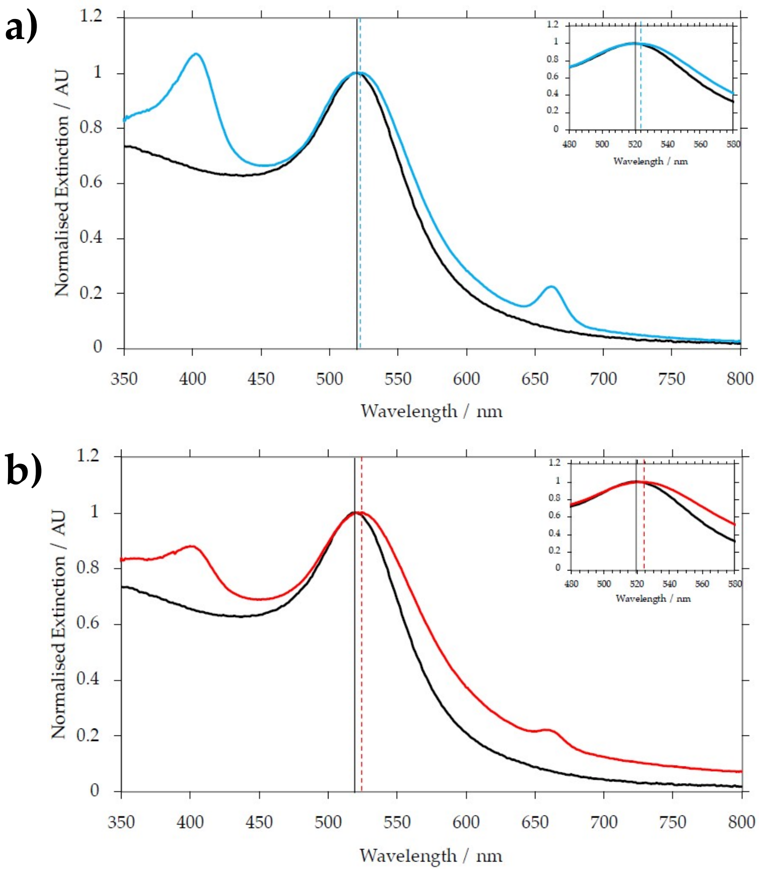

Figure S9. Normalised extinction spectra of citrate-AuNPs (black line) and **a) Gal-PEG$_3$-/ce6-PEG$_4$-AuNPs** (blue line); and **b) PEG$_3$-/ce6-PEG$_4$-AuNPs** (red line). Inset: Vertical black line represents extinction maximum for citrate-AuNPs, dotted lines represent extinction maxima for the functionalised AuNPs.

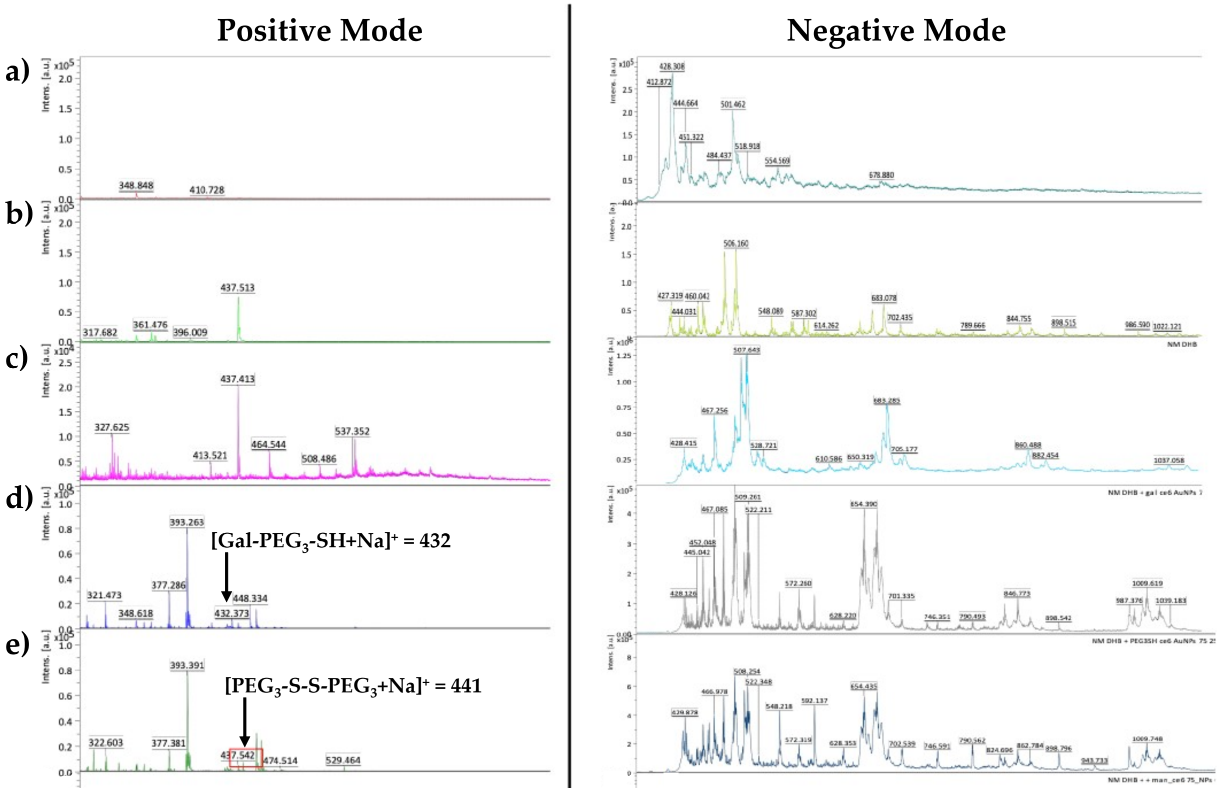

Figure S10. Positive (**left**) and negative (**right**) mode MALDI-TOF analysis of **Gal-PEG$_3$-/ce6-PEG$_4$-AuNPs**, with **a)** DHB matrix; **b)** DHB matrix with citrate-AuNPs; **c)** DHB matrix with ce6; **d)** DHB matrix with **Gal-PEG$_3$-/ce6-PEG$_4$-AuNPs**; and **e)** DHB matrix with **PEG$_3$-/ce6-PEG$_4$-AuNPs**.

2.3.2 Gal-PEG$_3$-AuNPs

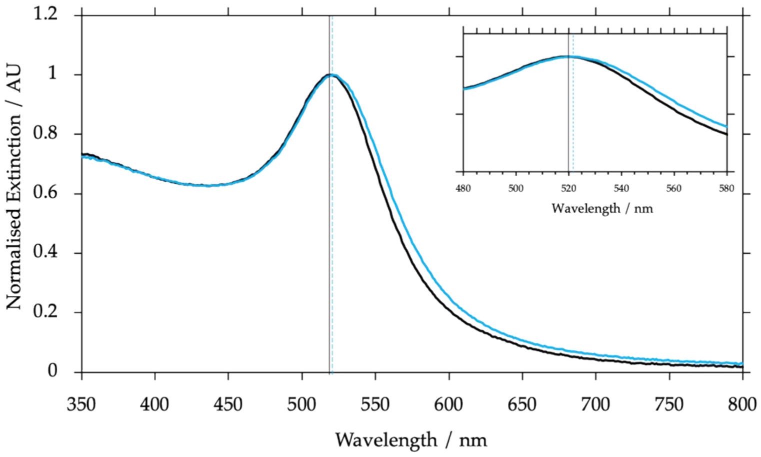

Figure S11. Normalised UV-Vis extinction spectra of citrate-AuNPs (black line) and **Gal-PEG$_3$-AuNPs** (blue line). Inset: Vertical black line represents extinction maximum for citrate-AuNPs, dotted blue line represent extinction maximum for the functionalised AuNPs.

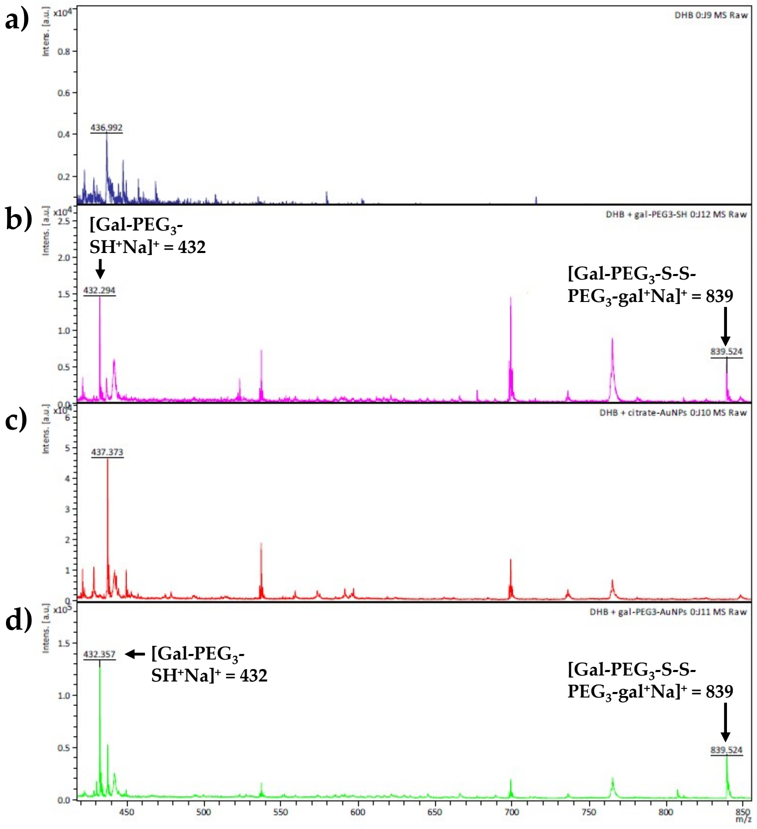

Figure S12. MALDI-TOF analysis of **Gal-PEG$_3$-AuNPs**. **a)** DHB matrix; **b)** DHB matrix with **Gal-PEG$_3$-SH**; **c)** DHB matrix with citrate-AuNPs; and **d)** DHB matrix with **Gal-PEG$_3$-AuNPs**.

2.4 Glycan uptake by cancer and non-cancer breast cell lines using glycan-/ce6-PEG$_4$-AuNPs

2.4.1 Cancer cell line: MDA-MB-231

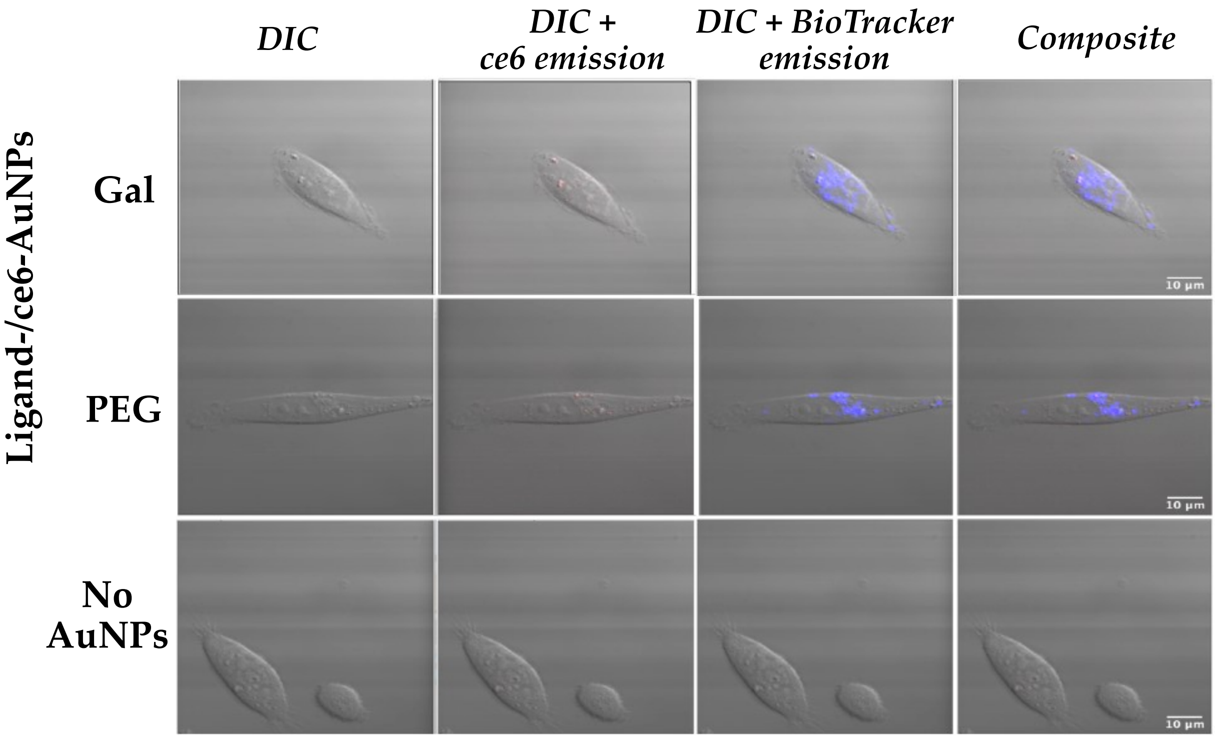

Figure S13. Example of processed confocal images from MDA-MB-231 treated with AuNPs: **Gal-PEG$_3$-/ce6-PEG$_4$-AuNPs** (**top**), **PEG$_3$-/ce6-PEG$_4$-AuNPs** (**middle**) and no AuNPs (**bottom**). Images are separated into DIC image, DIC image + fluorescence emission due to the ce6 on the uptaken AuNPs (shown in red), DIC image + emission from the BioTracker Orange dye (marker of acidic organelles, shown in blue) and composite, from left to right.

2.4.2 Cancer cell line: SK-BR-3

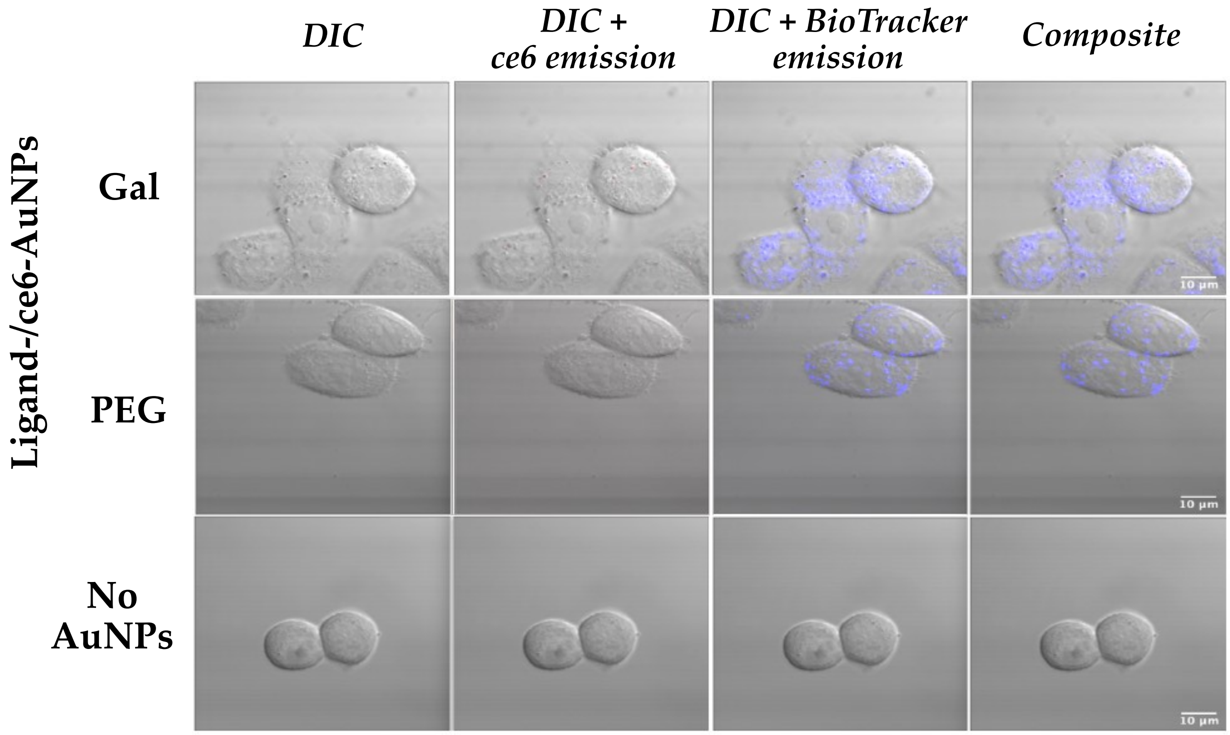

Figure S14. Example of processed confocal images from SK-BR-3 treated with AuNPs AuNPs: **Gal-PEG$_3$-/ce6-PEG$_4$-AuNPs** (**top**), **PEG$_3$-/ce6-PEG$_4$-AuNPs** (**middle**) and no AuNPs (**bottom**). Images are separated into DIC image, DIC image + fluorescence emission due to the ce6 on the uptaken AuNPs (shown in red), DIC image + emission from the BioTracker Orange dye (marker of acidic organelles, shown in blue) and composite, from left to right.

2.4.3 Healthy cell line: MCF-10A

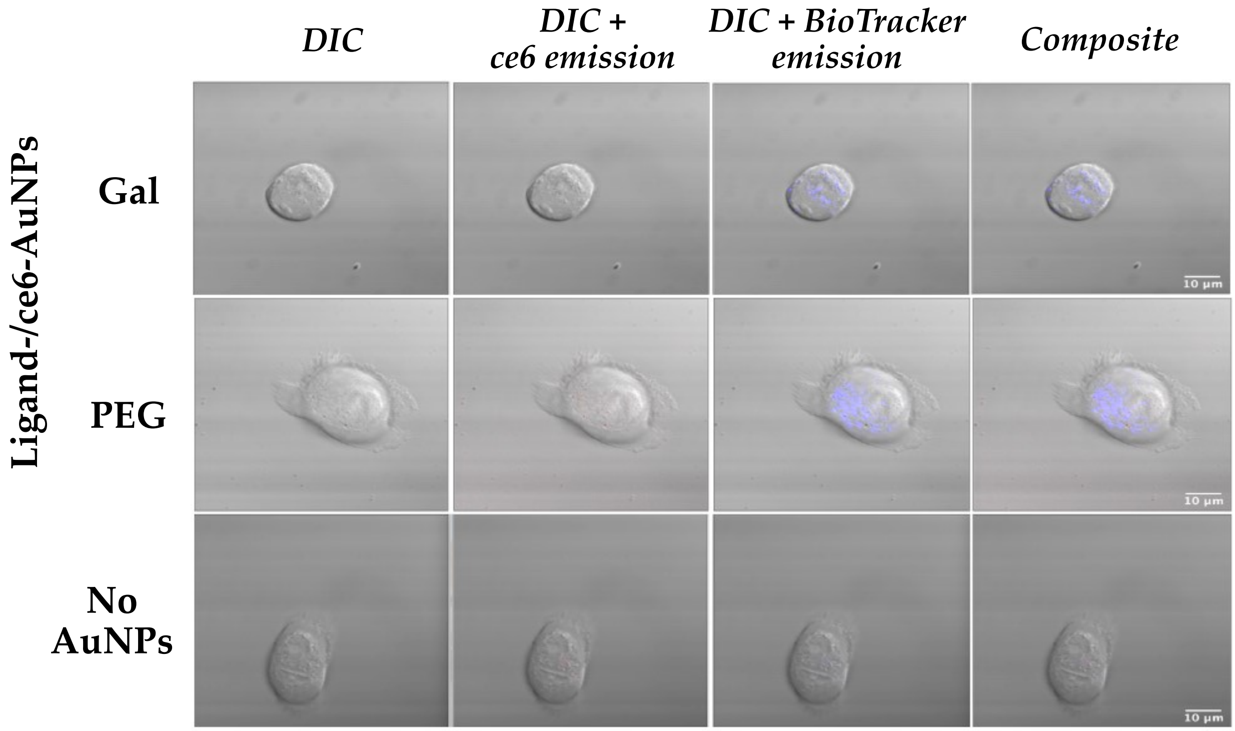

Figure S15. Example of processed confocal images from MCF-10A treated with AuNPs: **Gal-PEG$_3$-/ce6-PEG$_4$-AuNPs** (**top**), **PEG$_3$-/ce6-PEG$_4$-AuNPs** (**middle**) and no AuNPs (**bottom**). Images are separated into DIC image, DIC image + fluorescence emission due to the ce6 on the uptaken AuNPs (shown in red), DIC image + emission from the BioTracker Orange dye (marker of acidic organelles, shown in blue) and composite, from left to right.

2.5 Receptor uptake of Gal-PEG$_3$-/ce6-PEG$_4$-AuNPs by breast cancer cell lines

Table S2. Inhibitors of galactose binding protein expressed on breast cancer cell lines.

Inhibitor	***Target Receptors***
Canagliflozin	SGLT-1, - 2
33DFTG	Galectin-1, -3
WZB117	GLUT-1, -3, -4

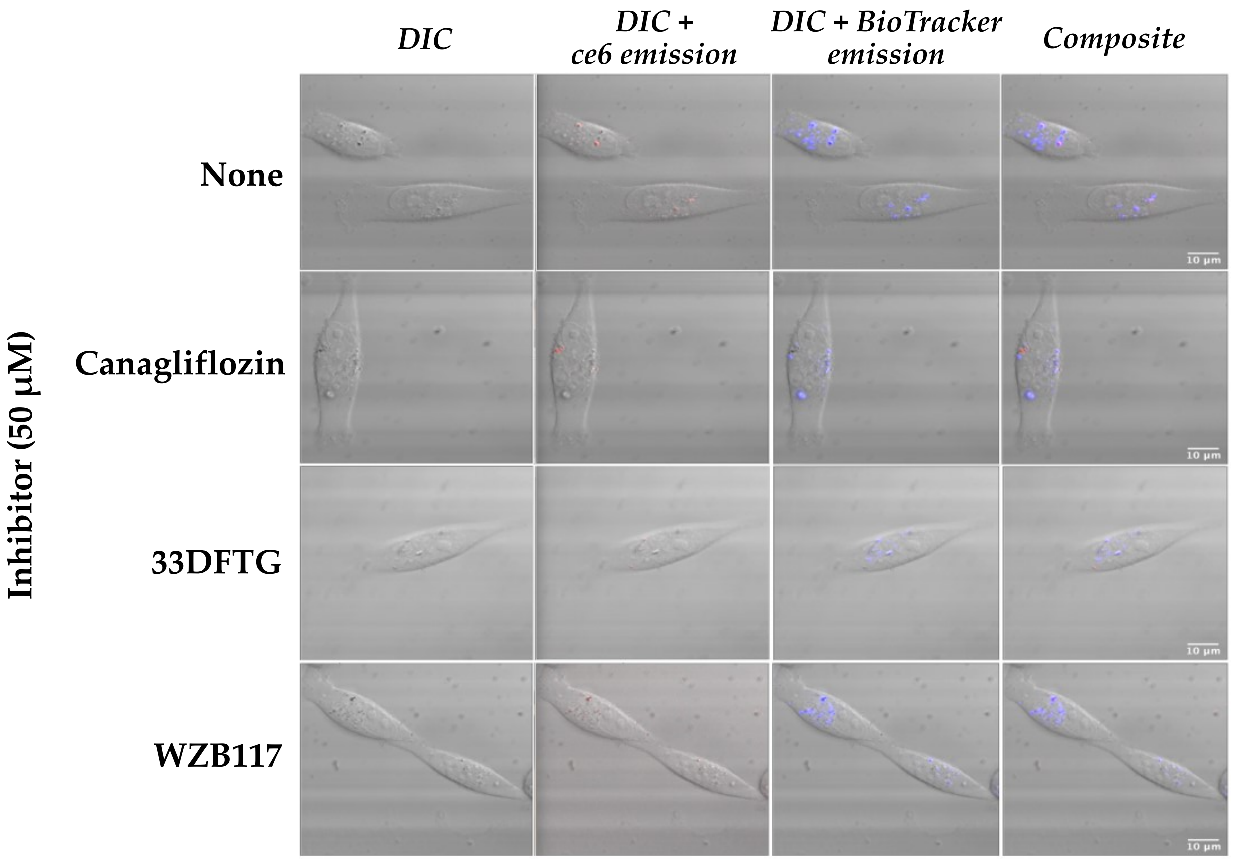

Figure S16. Example of processed confocal images for MDA-MB-231 protein binding inhibition to **Gal-PEG$_3$-/ce6-PEG$_4$-AuNPs**. No inhibitor, and presence of SGLT (canagliflozin), galectin (33DFTG), and GLUT (WZB117) inhibitors (top to bottom). Images are separated into DIC image, DIC image + fluorescence emission due to the ce6 on the uptaken **Gal-PEG$_3$-/ce6-PEG$_4$-AuNPs** (shown in red), DIC image + emission from the BioTracker Orange dye (marker of acidic organelles, shown in blue) and composite, from left to right.

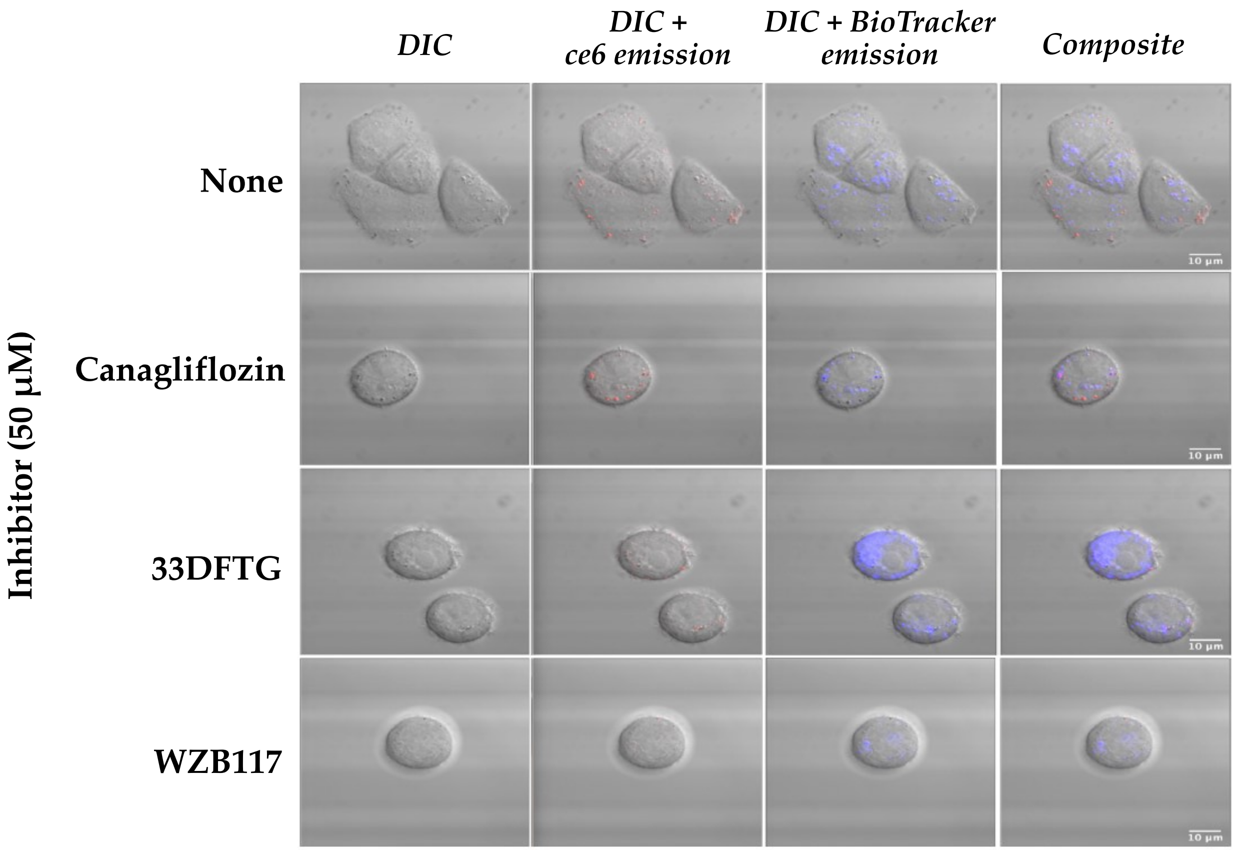

Figure S17. Example of processed confocal images for SK-BR-3 protein binding inhibition to **Gal-PEG$_3$-/ce6-PEG$_4$-AuNPs**. No inhibitor, and presence of SGLT (canagliflozin), galectin (33DFTG), and GLUT (WZB117) inhibitors (top to bottom). Images are separated into DIC image, DIC image + fluorescence emission due to the ce6 on the uptaken **Gal-PEG$_3$-/ce6-PEG$_4$-AuNPs** (shown in red), DIC image + emission from the BioTracker Orange dye (marker of acidic organelles, shown in blue) and composite, from left to right.

a) b)

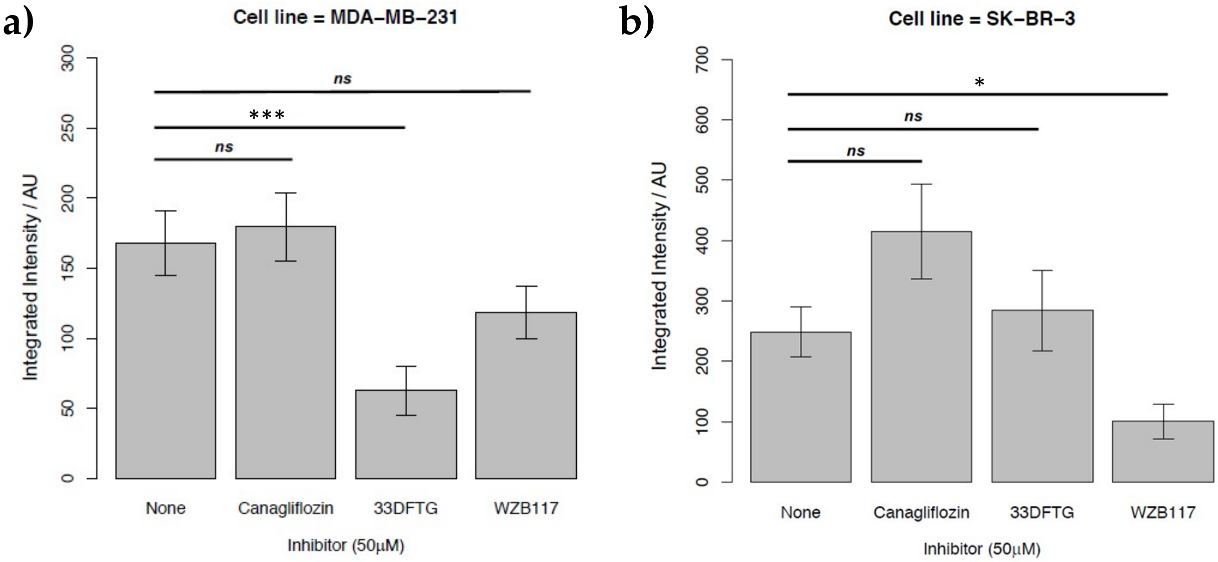

Figure S18. Quantitative analysis of confocal images from **Gal-PEG$_3$-/ce6-PEG$_4$-AuNPs** binding inhibition to **a)** MDA-MB-231 and **b)** SK-BR-3 glycan-binding proteins, showing the integrated intensity with the different inhibitors present: no inhibitor, SGLT (canagliflozin), galectin (33DFTG), and GLUT (WZB117) inhibitors, from left to right. Error bars = +/- SEM, n=7images. * = $p < 0.05$; ** = $p < 0.01$; *** = $p < 0.005$; ns = not significant (Welch two sample t-test).
